# Supplementary material for: Novel travel time aware metapopulation models and multi-layer waning immunity for late-phase epidemic and endemic scenarios
Source: PLoS Comput Biol. 2024 Dec 16;20(12):e1012630. doi: 10.1371/journal.pcbi.1012630 (PMC11684649; doi:10.1371/journal.pcbi.1012630)
Supplement: S1 Text — Fig A. Initialization of the Exposed compartment. For an arbitrary but fixed t0, individuals who got exposed in the left, red area will get symptoms or recover in the right, blue area. Table A. Parameters used to define our multi-layer waning immunity model of SECIRS-type. (PDF) [file pcbi.1012630.s001.pdf]

S1 Text: Full waning model equations and initialization

Local SECIRS-type model with waning immunity

In order to present the complete model equations, we define the most important parameters again. For more details, we refer to the subsection *A multi-layer waning immunity model of SECIRS-type.*

For the sake of simplicity, we drop the spatial-dependency superindex $*^{(k)}$. Let $\mu_{z_{j,i}}^{z_{j+1,i}}$ be the probability of transition from disease compartment $z_{j,i}$ to the worsened state $z_{j+1,i}$. Then, $1-\mu_{z_{j,i}}^{z_{j+1,i}}$ is the probability to recover from disease state $z_{j,i}$. Let further $T_{z_{j,i}}$ be the time in days an individual stays in a compartment $z_{j,i}$. Additionally, we define $N_i^{D^\perp}$ as the number of people from age group i which are not in the dead compartment.

Therefore, the systems of equations is given by

$$\begin{aligned}
\frac{dS_{N,i}}{dt} &= -S_{N,i}\rho_{N,i}\sum_{j=1}^{n}\phi_{i,j} \\
&\quad \frac{\xi_{I_{NS},j}(I_{NS,N,j}+I_{NS,PI,j}+I_{NS,II,j})+\xi_{I_{Sy},j}(I_{Sy,N,j}+I_{Sy,PI,j}+I_{Sy,II,j})}{N_j^{D^\perp}} \\
&\quad - v_{N,i}S_{N,i}+\frac{S_{PI,i}}{T_{W_{PI}}}, \qquad (1) \\
\frac{dE_{N,i}}{dt} &= S_{N,i}\rho_{N,i}\sum_{j=1}^{n}\phi_{i,j} \\
&\quad \frac{\xi_{I_{NS},j}(I_{NS,N,j}+I_{NS,PI,j}+I_{NS,II,j})+\xi_{I_{Sy},j}(I_{Sy,N,j}+I_{Sy,PI,j}+I_{Sy,II,j})}{N_j^{D^\perp}} \\
&\quad - \frac{E_{N,i}}{T_{E_i}}, \qquad (2) \\
\frac{dI_{NS,N,i}}{dt} &= \frac{E_{N,i}}{T_{E_i}}-\frac{I_{NS,N,i}}{T_{I_{NS,i}}}, \qquad (3) \\
\frac{dI_{NS,N,C,i}}{dt} &= -\frac{I_{NS,N,C,i}}{T_{I_{NS,i}}}, \qquad (4) \\
\frac{dI_{Sy,N,i}}{dt} &= \frac{\mu_{I_{NS,N,i}}^{I_{Sy,N,i}}}{T_{I_{NS,i}}}I_{NS,N,i}-\frac{I_{Sy,N,i}}{T_{I_{Sy,i}}}, \qquad (5) \\
\frac{dI_{Sy,N,C,i}}{dt} &= \frac{\mu_{I_{NS,N,i}}^{I_{Sy,N,i}}}{T_{I_{NS,i}}}I_{NS,N,C,i}-\frac{I_{Sy,N,C,i}}{T_{I_{Sy,i}}}, \qquad (6) \\
\frac{dI_{Sev,N,i}}{dt} &= \frac{\mu_{I_{Sy,N,i}}^{I_{Sev,N,i}}}{T_{I_{Sy,i}}}\Big(I_{Sy,N,i}+I_{Sy,N,C,i}\Big)-\frac{I_{Sev,N,i}}{T_{I_{Sev,i}}}, \qquad (7) \\
\frac{dI_{Cr,N,i}}{dt} &= \frac{\mu_{I_{Sev,N,i}}^{I_{Cr,N,i}}}{T_{I_{Sev,i}}}I_{Sev,N,i}-\frac{I_{Cr,N,i}}{T_{I_{Cr,i}}}, \qquad (8) \\
\frac{dD_{N,i}}{dt} &= \frac{\mu_{I_{Cr,N,i}}^{D_{N,i}}}{T_{I_{Cr,i}}}I_{Cr,N,i}, \qquad (9) \\
\frac{dS_{PI,i}}{dt} &= -S_{PI,i}\rho_{PI,i}\sum_{j=1}^{n}\phi_{i,j}
\end{aligned}$$

$$\frac{\xi_{I_{NS,j}}(I_{NS,N,j}+I_{NS,PI,j}+I_{NS,II,j})+\xi_{I_{Sy},j}(I_{Sy,N,j}+I_{Sy,PI,j}+I_{Sy,II,j})}{N_j^{D^{\perp}}}$$

$$-v_{PI,i}S_{PI,i}+\frac{\mathcal{T}_{PI,i}}{T_{\mathcal{T}_{PI,i}}}-\frac{S_{PI,i}}{T_{W_{PI}}}+\frac{S_{II,i}}{T_{W_{II}}}, \tag{10}$$

$$\frac{dE_{PI,i}}{dt}=S_{PI,i}\rho_{PI,i}\sum_{j=1}^{n}\phi_{i,j}$$

$$\frac{\xi_{I_{NS,j}}(I_{NS,N,j}+I_{NS,PI,j}+I_{NS,II,j})+\xi_{I_{Sy},j}(I_{Sy,N,j}+I_{Sy,PI,j}+I_{Sy,II,j})}{N_j^{D^{\perp}}}$$

$$-\frac{E_{PI,i}}{T_{E_i}}, \tag{11}$$

$$\frac{dI_{NS,PI,i}}{dt}=\frac{E_{PI,i}}{T_{E_i}}-\frac{I_{NS,PI,i}}{\kappa T_{I_{NS,i}}}, \tag{12}$$

$$\frac{dI_{NS,PI,C,i}}{dt}=-\frac{I_{NS,PI,C,i}}{\kappa T_{I_{NS,i}}}, \tag{13}$$

$$\frac{dI_{Sy,PI,i}}{dt}=\frac{\mu_{I_{NS,PI,i}}^{I_{Sy,PI,i}}}{\kappa T_{I_{NS,i}}}I_{NS,PI,i}-\frac{I_{Sy,PI,i}}{\kappa T_{I_{Sy,i}}}, \tag{14}$$

$$\frac{dI_{Sy,PI,C,i}}{dt}=\frac{\mu_{I_{NS,PI,i}}^{I_{Sy,PI,i}}}{\kappa T_{I_{NS,i}}}I_{NS,PI,C,i}-\frac{I_{Sy,PI,C,i}}{\kappa T_{I_{Sy,i}}}, \tag{15}$$

$$\frac{dI_{Sev,PI,i}}{dt}=\frac{\mu_{I_{Sy,PI,i}}^{I_{Sev,PI,i}}}{\kappa T_{I_{Sy,i}}}\Big(I_{Sy,PI,i}+I_{Sy,PI,C,i}\Big)-\frac{I_{Sev,PI,i}}{T_{I_{Sev,i}}}, \tag{16}$$

$$\frac{dI_{Cr,PI,i}}{dt}=\frac{\mu_{I_{Sev,PI,i}}^{I_{Cr,PI,i}}}{T_{I_{Sev,i}}}I_{Sev,PI,i}-\frac{I_{Cr,PI,i}}{T_{I_{Cr,i}}}, \tag{17}$$

$$\frac{dD_{PI,i}}{dt}=\frac{\mu_{I_{Cr,PI,i}}^{D_{PI,i}}}{T_{I_{Cr,i}}}I_{Cr,PI,i}, \tag{18}$$

$$\frac{dS_{II,i}}{dt}=-S_{II,i}\rho_{II,i}\sum_{j=1}^{n}\phi_{i,j}$$

$$\frac{\xi_{I_{NS,j}}(I_{NS,N,j}+I_{NS,PI,j}+I_{NS,II,j})+\xi_{I_{Sy},j}(I_{Sy,N,j}+I_{Sy,PI,j}+I_{Sy,II,j})}{N_j^{D^{\perp}}}$$

$$-v_{II,i}S_{II,i}-\frac{S_{II,i}}{T_{W_{II}}}+\frac{\mathcal{T}_{II,i}}{T_{\mathcal{T}_{II,i}}}, \tag{19}$$

$$\frac{dE_{II,i}}{dt}=S_{II,i}\rho_{II,i}\sum_{j=1}^{n}\phi_{i,j}$$

$$\frac{\xi_{I_{NS,j}}(I_{NS,N,j}+I_{NS,PI,j}+I_{NS,II,j})+\xi_{I_{Sy},j}(I_{Sy,N,j}+I_{Sy,PI,j}+I_{Sy,II,j})}{N_j^{D^{\perp}}}$$

$$-\frac{E_{II,i}}{T_{E_i}}, \tag{20}$$

$$\frac{dI_{NS,II,i}}{dt}=\frac{E_{II,i}}{T_{E_i}}-\frac{I_{NS,II,i}}{\kappa T_{I_{NS,i}}}, \tag{21}$$

$$\frac{dI_{NS,II,C,i}}{dt}=-\frac{I_{NS,II,C,i}}{\kappa T_{I_{NS,i}}}, \tag{22}$$

$$\frac{dI_{Sy,II,i}}{dt} = \frac{\mu^{I_{Sy,II,i}}_{I_{NS,II,i}}}{\kappa T_{I_{NS,i}}} I_{NS,II,i} - \frac{I_{Sy,II,i}}{\kappa T_{I_{Sy,i}}}, \tag{23}$$

$$\frac{dI_{Sy,II,C,i}}{dt} = \frac{\mu^{I_{Sy,II,i}}_{I_{NS,II,i}}}{\kappa T_{I_{NS,i}}} I_{NS,II,C,i} - \frac{I_{Sy,II,C,i}}{\kappa T_{I_{Sy,i}}}, \tag{24}$$

$$\frac{dI_{Sev,II,i}}{dt} = \frac{\mu^{I_{Sev,II,i}}_{I_{Sy,II,i}}}{\kappa T_{I_{Sy,i}}} \Big(I_{Sy,II,i} + I_{Sy,II,C,i} \Big) - \frac{I_{Sev,II,i}}{T_{I_{Sev,i}}}, \tag{25}$$

$$\frac{dI_{Cr,II,i}}{dt} = \frac{\mu^{I_{Cr,II,i}}_{I_{Sev,II,i}}}{T_{I_{Sev,i}}} I_{Sev,II,i} - \frac{I_{Cr,II,i}}{T_{I_{Cr,i}}}, \tag{26}$$

$$\frac{dD_{II,i}}{dt} = \frac{\mu^{D_{II,i}}_{I_{Cr,II,i}}}{T_{I_{Cr,i}}} I_{Cr,II,i}, \tag{27}$$

$$\begin{aligned} \frac{d\mathcal{T}_{PI,i}}{dt} &= -\frac{\mathcal{T}_{PI,i}}{T_{\mathcal{T}_{PI,i}}} + \frac{\mu^{\mathcal{T}_{PI,i}}_{I_{NS,N,i}}}{T_{I_{NS,i}}} (I_{NS,N,i} + I_{NS,N,C,i}) \\ &+ \frac{1 - \mu^{I_{Sev,N,i}}_{I_{Sy,N,i}}}{T_{I_{Sy,i}}} (I_{Sy,N,i} + I_{Sy,N,C,i}) \\ &+ \frac{1 - \mu^{I_{Cr,N,i}}_{I_{Sev,N,i}}}{T_{I_{Sev,i}}} I_{Sev,N,i} + \frac{1 - \mu^{D_{N,i}}_{I_{Cr,N,i}}}{T_{I_{Cr,i}}} I_{Cr,N,i} + v_{N,i} S_{N,i}, \end{aligned} \tag{28}$$

$$\begin{aligned} \frac{d\mathcal{T}_{II,i}}{dt} &= -\frac{\mathcal{T}_{II,i}}{T_{\mathcal{T}_{II,i}}} + \frac{1 - \mu^{I_{Sy,II,i}}_{I_{NS,PI,i}}}{\kappa T_{I_{NS,i}}} (I_{NS,PI,i} + I_{NS,PI,C,i}) \\ &+ \frac{1 - \mu^{I_{Sev,PI,i}}_{I_{Sy,PI,i}}}{\kappa T_{I_{Sy,i}}} (I_{Sy,PI,i} + I_{Sy,PI,C,i}) \\ &+ \frac{1 - \mu^{I_{Cr,PI,i}}_{I_{Sev,PI,i}}}{T_{I_{Sev,i}}} I_{Sev,PI,i} + \frac{1 - \mu^{D_{PI,i}}_{I_{Cr,PI,i}}}{T_{I_{Cr,i}}} I_{Cr,PI,i} \\ &+ \frac{1 - \mu^{I_{Sy,II,i}}_{I_{NS,II,i}}}{\kappa T_{I_{NS,i}}} (I_{NS,II,i} + I_{NS,II,C,i}) + \frac{1 - \mu^{I_{Sev,II,i}}_{I_{Sy,II,i}}}{\kappa T_{I_{Sy,i}}} (I_{Sy,II,i} + I_{Sy,II,C,i}) \\ &+ \frac{1 - \mu^{I_{Cr,II,i}}_{I_{Sev,II,i}}}{T_{I_{Sev,i}}} I_{Sev,II,i} + \frac{1 - \mu^{D_{II,i}}_{I_{Cr,II,i}}}{T_{I_{Cr,i}}} I_{Cr,II,i} + v_{PI,i} S_{PI,i} + v_{II,i} S_{II,i}. \end{aligned} \tag{29}$$

An explanation of all used parameters for defining the model in Equations (1) - (29) is provided in Table A.

Initialization of the introduced compartmental models.

For simplicity, we explain our approach for one age group. It naturally transfers to all other age groups. To determine the initial states for the individual compartments of our model, we use the German case numbers published by the Robert Koch-Institute [1]. Note that any other reporting on the considered geography could be used. In our use case, we perform the initialization on county level. The following descriptions is however possible for any granularity, assuming that the data is available at this level. The initial idea for our approach to use the reported case data as initial states for each compartment was given in [2]. We state that the use of time spans or rates depending on the start **and** target compartment, e.g., T_C^I and T_C^R may have the unwanted effect that μ_C^R no longer represents a probability, as initially assumed and modeled. We thus

Table A. Parameters used to define our multi-layer waning immunity model of SECIRS-type; Eq (1) - (29).

Parameter	Description
$\phi_{i,j}$	Daily contact rate between two age groups i and j.
$\rho_{0,N}$	Baseline transmission risk for people located in the naive susceptible compartments
k	Seasonality parameter
$N_i^{D^\perp}$	People from age group i which have not died during simulation.
$\mu_{z_1}^{z_2}$	Probability of transition from compartment z_1 to z_2.
T_{z_1}	Time in days an individual stays in a compartment z_1.
$\xi_{I_{NS},i}$	Proportion of asymptomatic infectious people who are not isolated.
$\xi_{I_{Sy},i}$	Proportion of symptomatic infectious people who are not isolated.
κ	Reduction factor for time spans of asymptomatic and symptomatic infections of individuals with partial or improved immunity.
$p_{E_{PI}}$	Effectiveness of partial immunity protection against infection.
$p_{I_{Sy,PI}}$	Effectiveness of partial immunity protection against symptomatic infection.
$p_{I_{Sev,PI}}$	Effectiveness of partial immunity protection against hospitalization.
$p_{I_{Cr,PI}}$	Effectiveness of partial immunity protection against ICU treatment.
$p_{D_{PI}}$	Effectiveness of partial immunity protection against death.
$p_{E_{II}}$	Effectiveness of improved immunity protection against infection.
$p_{I_{Sy,II}}$	Effectiveness of improved immunity protection against symptomatic infection.
$p_{I_{Sev,II}}$	Effectiveness of improved immunity protection against hospitalization.
$p_{I_{Cr,II}}$	Effectiveness of improved immunity protection against ICU treatment.
$p_{D_{II}}$	Effectiveness of improved immunity protection against death.
$T_{W_{PI}}$	Rate of waning immunity of susceptible who are located in the partial immunity state.
$T_{W_{II}}$	Rate of waning immunity of susceptible who are located in the improved immunity state.
v_N	Vaccination rate of people with naive immunity.
v_{PI}	Vaccination rate of susceptible with partial immunity.
v_{II}	Vaccination rate of people with improved immunity.

reduce the description to using $T_C = T_C^I = T_C^R$ and present this approach with additional adjustments to our model.

The idea behind the procedure is to extrapolate the reported numbers of cases only from the model stay times and transition probabilities. We refer to the number of cases (in the particular age group) as $\sum^C(t)$. Similarly, the number of deaths reported (in the particular age group) will be denoted $\sum^D(t)$. Following [2], we make the assumption that the number of confirmed cases is proportional to the number of symptomatic cases. Furthermore, we use the (accumulated) numbers of partial, full and booster vaccinations (in the particular age group) up to day t [3] to determine the vaccinations in the particular immunity layers. We use reported partial vaccinations $\sum^{V_N}(t)$ for the naive layer, the reported full vaccinations $\sum^{V_{PI}}(t)$ for the partial immunity layer and all reported booster vaccinations $\sum^{V_{II}}(t)$ for the improved immunity layer. Assuming a

nontrivial but constant underdetection factor for the initialization phase, we define a factor θ accounting for the estimated dark figure. The estimated number of reported and unreported symptomatic infections on day t is then given by

$$\widehat{\Sigma}^C(t) = \theta \Sigma^C(t). \tag{30}$$

Furthermore, for simplicity, we assume that detection (or, more general, inclusion into $\widehat{\Sigma}^C(t)$) happens at the time of developing systems. In a generalized approach, an additional shift $\widehat{\Sigma}^C(t + \delta_T)$, with $\delta_T > 0$ a testing or reporting delay, could be added.

As already mentioned in the introduction of the model, we divide the population into three different immunity levels. In [4], an age-resolved breakdown of the German population into four different immunity levels is given. In this study exposure to the virus is defined as a survived infection or vaccination with a corresponding immune response. The classification in four groups is based on the degree of exposure: people with no confirmed exposures, people with one or two confirmed exposures, people with exactly three confirmed exposures and those surpassing three confirmed exposures. Since the proportion of individuals with zero confirmed exposures to date is extremely small, we aggregate them with the group of one or two exposures. Thus, the division takes place into a subpopulation N_N with zero, one or two exposures, a subpopulation N_{PI} with exactly three exposures, and a subpopulation N_{II} with more than three exposures. From the results of [4], we can estimate the immunity of the total population at hand. Next, we explain how we use the reported numbers to estimate the distribution of the population inside the immunity layers.

To determine the initial state of individuals in the exposed state as shown, we proceed as visualized in Fig. A.Based on our defined transition times, individuals that get exposed at t_0 develop symptoms at $t_0 + T_E + T_{I_{NS}}$ (on average) – and would have been reported or, more generally, included in $\widehat{\Sigma}^C(t_0 + T_E + T_{I_{NS}})$. Individuals who are still exposed at t_0 but become pre- or asymptomatic shortly after that, either develop symptoms by time $t_0 + T_{I_{NS}}$ or will have recovered by then (i.e., will have been included in $\widehat{\Sigma}^C(t_0) + T_{I_{NS}}$). Thus, the total number of exposed at t_0 computes as

$$\sum_{m=1}^{3} E_{Y_m}(t_0) = \left(\frac{1}{\mu_{I_{NS,Y_m}}^{I_{Sy,Y_m}}} (\widehat{\Sigma}^C(t_0 + T_E + T_{I_{NS}}) - \widehat{\Sigma}^C(t_0 + T_{I_{NS}})) \right), \tag{31}$$

where $Y_m \in \{N, PI, II\}$ represents any immunity layer.

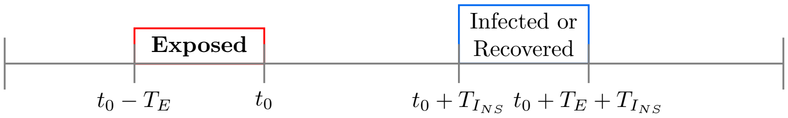

Fig A. Initialization of the Exposed compartment. For an arbitrary but fixed t_0, individuals who got exposed in the left, red area will get symptoms or recover in the right, blue area.

We determine the initial number of exposed individuals for any immunity layer Y_m by a uniform distribution to the three immunity layers, i.e.,

$$E_{Y_m}(t_0) = \frac{N_{Y_m}(t_0)}{\sum_{j=1}^{3} N_{Y_j}(t_0)} \left(\frac{1}{\mu_{I_{NS,Y_m}}^{I_{Sy,Y_m}}} (\widehat{\Sigma}^C(t_0 + T_E + T_{I_{NS}}) - \widehat{\Sigma}^C(t_0 + T_{I_{NS}})) \right). \tag{32}$$

We can adapt this scheme to all other compartments. Therefore, the other equations are

given by

$$
\begin{aligned}
I_{NS,Y_m}(t_0) &= \frac{N_{Y_m}(t_0)}{\sum_{j=1}^{3} N_{Y_j}(t_0)} \left(\frac{1}{\mu_{I_{NS,Y_m}}^{I_{Sy,Y_m}}} (\widehat{\Sigma}^C(t_0 + T_{I_{NS}}) - \widehat{\Sigma}^C(t_0)) \right), \\
I_{Sy,Y_m}(t_0) &= \frac{N_{Y_m}(t_0) p_{I_{Sy,Y_m}}}{\sum_{j=1}^{3} N_{Y_j}(t_0) p_{I_{Sy,Y_j}}} \left(\widehat{\Sigma}^C(t_0) - \widehat{\Sigma}^C(t_0 - T_{I_{Sy}}) \right), \\
I_{Sev,Y_m}(t_0) &= \frac{N_{Y_m}(t_0) p_{I_{Sev,Y_m}}}{\sum_{j=1}^{3} N_{Y_j}(t_0) p_{I_{Sev,Y_j}}} \left(\mu_{I_{Sy,Y_N}}^{I_{Sev,Y_N}} (\widehat{\Sigma}^C(t_0 - T_{I_{Sy}}) - \widehat{\Sigma}^C(t_0 - T_{I_{Sy}} - T_{I_{Sev}}) \right).
\end{aligned} \tag{33}
$$

Note that for symptomatic and subsequent infection states, we use a nonuniform distribution, taking into account the effectiveness of protection of the different immunity layers against severe courses of the disease. The group $I_{Cr,Y_m}(t_0)$ is intentionally left out here, as it is initialized directly with daily ICU reports from [5].

To calculate the total deaths per immunity layer, it is essential to track the complete historical data on layer sizes and fatalities. In our model, however, we only model the deaths during the simulation phase and initialize

$$
D_{Y_m}(t_0) = 0. \tag{34}
$$

Previous death numbers have to be added to the simulation result in post-processing to see the overall number of deaths.

Finally, we need to determine the number of individuals in the temporary immunity and the susceptible compartments at t_0. In order to calculate the number of temporarily immune people, we need to estimate the number of recently recovered $R^R_{\hat{Y}_m}(t) := \frac{N_{\hat{Y}_m}(t_0)}{\sum_j N_{Y_j}(t_0)} \left(\widehat{\Sigma}^C(t) - \widehat{\Sigma}^C(t - T_{\mathcal{I}_{\hat{Y}_m}}) \right)$, $\hat{Y}_m \in \{PI, II\}$, and recently vaccinated $R^V_{\hat{Y}_m} := \widehat{\Sigma}^{V_{\hat{Y}_m}}(t) - \widehat{\Sigma}^{V_{\hat{Y}_m}}(t - T_{\mathcal{I}_{\hat{Y}_m}})$, $\hat{Y}_m \in \{PI, II\}$, individuals within the last $T_{\mathcal{I}_{\hat{Y}_m}}$ days, such that

$$
\mathcal{I}_{\hat{Y}_m}(t) = \min \left\{ N_{\hat{Y}_m}, \max \left\{ 0, R^V_{\hat{Y}_m} + R^R_{\hat{Y}_m}(t) - \sum_m \left(I_{Sy,\hat{Y}_m}(t) + I_{Sev,\hat{Y}_m}(t) + I_{Cr,\hat{Y}_m}(t) \right) \right\} \right\}. \tag{35}
$$

The number of individuals in the susceptible compartment results directly from the remaining population in each layer that has not yet been taken into account in the other compartments.

References

1. Robert Koch-Institut. SARS-CoV-2 Infektionen in Deutschland; 2023. Zenodo. Available from: https://doi.org/10.5281/zenodo.7814948.

2. Koslow W, Kühn MJ, Binder S, Klitz M, Abele D, Basermann A, et al. Appropriate relaxation of non-pharmaceutical interventions minimizes the risk of a resurgence in SARS-CoV-2 infections in spite of the Delta variant. PLOS Computational Biology. 2022;18(5):e1010054. doi:10.1371/journal.pcbi.1010054.

3. Digitales Impfquotenmonitoring zur COVID-19-Impfung; 2023. Available from: https://www.rki.de/DE/Content/InfAZ/N/Neuartiges_Coronavirus/Daten/Impfquoten-Tab.html.

4. Lange B, Jäger V, Rücker V, Hassenstein M, Harries M, Berner R, et al.. Interimsanalyse des IMMUNEBRIDGE-Projektes zur Kommunikation von vorläufigen Ergebnissen an die Modellierungskonsortien der BMBF-geförderten Modellierungsplattform; 2022. Available from: https://doi.org/10.5281/zenodo.6968574.

5. Deutsche Interdisziplinäre Vereinigung für Intensiv- und Notfallmedizin (DIVI). DIVI Intensivregister Tagesreport; 2022. Available from: https://www.divi.de/divi-intensivregister-tagesreport-archiv.
