# Supplementary material for: Novel travel time aware metapopulation models and multi-layer waning immunity for late-phase epidemic and endemic scenarios
Source: PLoS Comput Biol. 2024 Dec 16;20(12):e1012630. doi: 10.1371/journal.pcbi.1012630 (PMC11684649; doi:10.1371/journal.pcbi.1012630)
Supplement: S1 Fig — The impact of each parameter is represented by a logarithmic scaled bar where the length represents the positive and negative influence. (PDF) [file pcbi.1012630.s002.pdf]

S1 Fig. Sensitivity Analysis

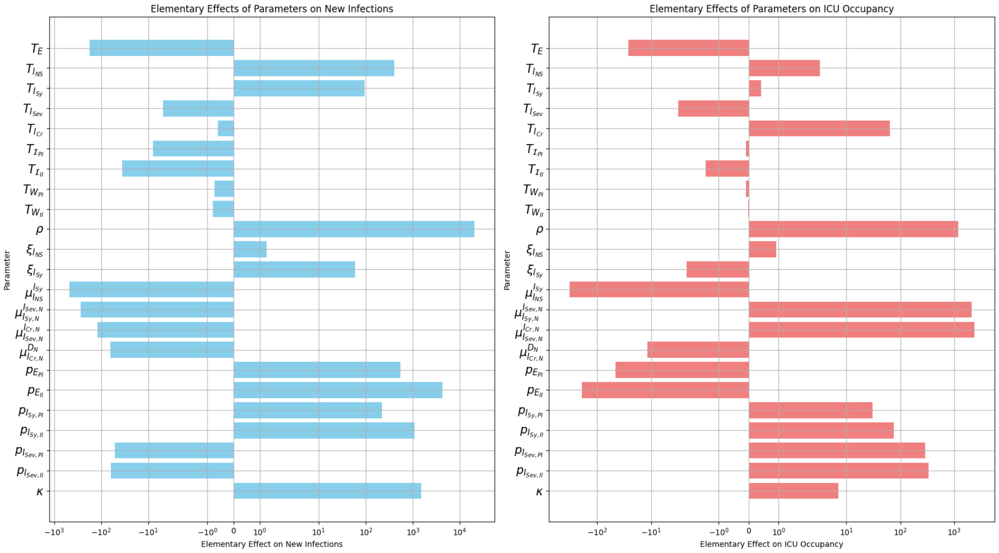

Fig A. Results of the sensitivity analysis showing the Elementary Effects for the model parameters with respect to the daily new infections (left) and ICU occupancy (right). The impact of each parameter is represented by a logarithmic scaled bar where the length represents the positive and negative influence.
