# Supplementary material for: Novel travel time aware metapopulation models and multi-layer waning immunity for late-phase epidemic and endemic scenarios
Source: PLoS Comput Biol. 2024 Dec 16;20(12):e1012630. doi: 10.1371/journal.pcbi.1012630 (PMC11684649; doi:10.1371/journal.pcbi.1012630)
Supplement: S2 Fig — Predicted viral load (LOESS regression) per sample location and sorted by federal state as obtained by [69]. (PDF) [file pcbi.1012630.s003.pdf]

# S2 Fig. AMELAG data by federal state.

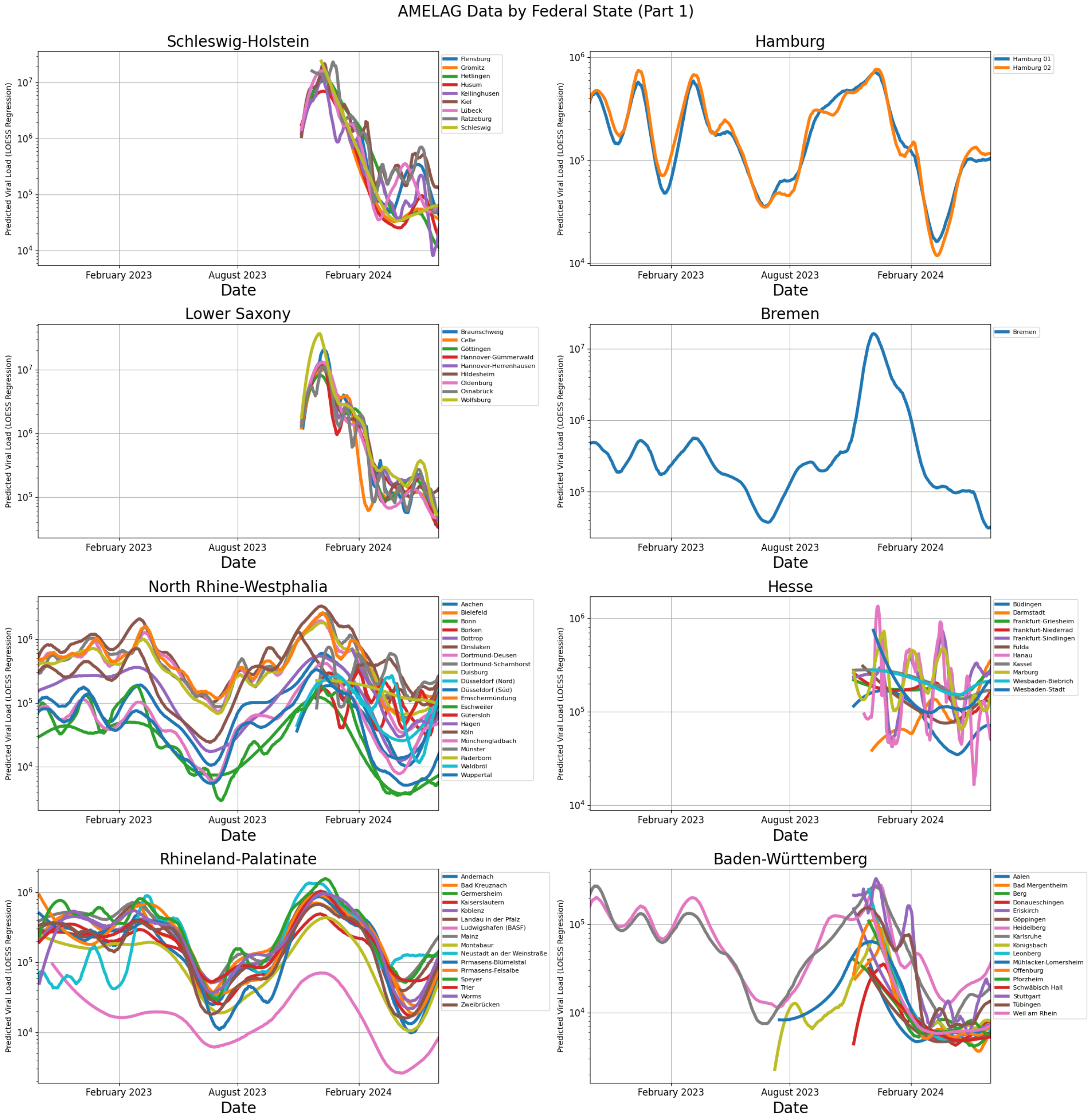


**Fig A. Recorded AMELAG wastewater data by federal state.** Predicted viral load (LOESS regression) per sample location and sorted by federal state as obtained by [1].

## References

1. Robert Koch-Institut F. Abwassersurveillance AMELAG; 2024. Available from: `https://doi.org/10.5281/zenodo.13683260`.

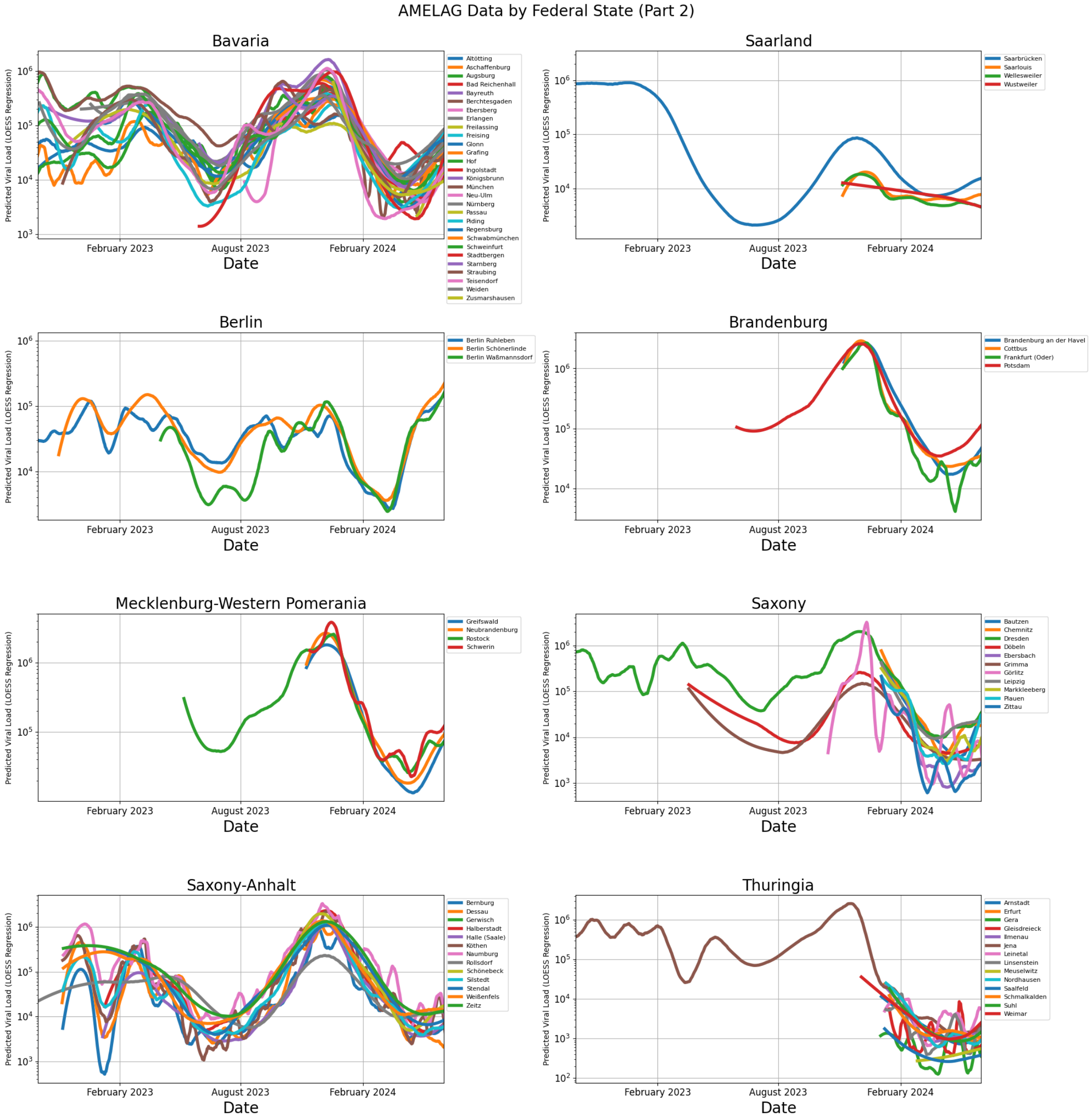


**Fig B. Recorded AMELAG wastewater data by federal state (cont'd).** Predicted viral load (LOESS regression) per sample location and sorted by federal state as obtained by [1].
