# Supplementary material for: Novel travel time aware metapopulation models and multi-layer waning immunity for late-phase epidemic and endemic scenarios
Source: PLoS Comput Biol. 2024 Dec 16;20(12):e1012630. doi: 10.1371/journal.pcbi.1012630 (PMC11684649; doi:10.1371/journal.pcbi.1012630)
Supplement: Attachment — Submitted filename: review.pdf [file pcbi.1012630.s004.pdf]

# Review on the manucript: Novel travel time aware metapopulation models: A combination with multi-layer waning immunity to assess late-phase epidemic and endemic scenarios

In this manuscript the authors studied a graph-ODE type epidemic model incorporating additional traffic contacts to non-travel contacts as well as immunity waning, designed for the disease dynamics across Germany's regions. The paper is well written and the models are described in detail. I have some comments that need to be addressed before I can recommend the paper for publication.

1. The authors claim that their model can be adapted to other infectious diseases beyond SARS-CoV-2. The travel contact model does not always obey (2)-(7). For instance, seasonality is not taken into account in the model. In addition, travel contact structure changes significantly during summer vacation, compared to regular days.
2. The authors assume that the number of contacts in non-traffic locations is independent of the additional contacts during travel. The authors have to justify such a strong assumption.
3. Given that the interest is in disease dynamics across Germany, the authors neglect the importation risk from different areas outside of Germany. Please comment on this.
4. The model is an SEIR-type model with immunity waning. I suggest to replace SECIRS by SEIRS to avoid any misleading to the reader.
5. The model lacks of the literature on immunity waning. It is important that the authors discuss how immunity wanes with respect to SARS-CoV-2 but also including latest research on modes of waning.
6. Given the high complexity level of the proposed model, the authors need to specify in what way their model improve upon traditional models. For instance, they need to discuss how their model behave with respect to models with no immunity waning, given a few months time frame.
7. Later, the authors compared their model with the one given in [17]. From Fig. 13, the epidemic shape shows different patterns. The robustness of this result is not addressed by the paper. Also, such a result was obtained using a fictional outbreak scenario. The authors have to clarify how these fictional data were obtained as well as how robust is their result.
8. As the model used is highly parameterized, a sensitivity analysis is needed to identify the parameters that have the most impact on the disease dynamics.
9. It is known that a large portion of transmissions happen before symptoms onset. The introduction of a presymptomatic compartment would be of great interest.
10. Some new parameters appear in Table 3: $p_{H_{PI}}$ and $p_{H_v}$! What are those parameters? I couldn't see them in the model definition.
11. The authors claim that the parameter values used for Omicron and in Table 3 are justified from the literature. The authors said to refer to [34,35], however, I couldn't find where the values of the exposure and infectious periods of $\approx 0.6$ come from.

**Minor comments:**
Legend in Fig 2. show $\phi = 9.1$ and $\phi = 9.05$. Please correct these legends.
Line 184. j and k should be written in mathematical style. Check the whole manuscript.

Line 208: An important ...
Line 432: ”is number”!
...
