# Supplementary material for: Novel travel time aware metapopulation models and multi-layer waning immunity for late-phase epidemic and endemic scenarios
Source: PLoS Comput Biol. 2024 Dec 16;20(12):e1012630. doi: 10.1371/journal.pcbi.1012630 (PMC11684649; doi:10.1371/journal.pcbi.1012630)
Supplement: Attachment — Submitted filename: Zunker_et_al_Mobility_aware___answer_to_reviewers.pdf [file pcbi.1012630.s005.pdf]

Reviewer #1

In this manuscript the authors studied a graph-ODE type epidemic model incorporating additional traffic contacts to non-travel contacts as well as immunity waning, designed for the disease dynamics across Germany's regions. The paper is well written and the models are described in detail. I have some comments that need to be addressed before I can recommend the paper for publication.
Answer: We thank the reviewer for the effort of a detailed review of our paper. We have addressed all the points mentioned by the reviewer and provide detailed answers in the following. Changes in the manuscript are highlighted accordingly.

1. *The authors claim that their model can be adapted to other infectious diseases beyond SARS-CoV-2. The travel contact model does not always obey (2)-(7). For instance, seasonality is not taken into account in the model. In addition, travel contact structure changes significantly during summer vacation, compared to regular days.*

   Answer: We actually feel that the reviewer made two particular points with this comment. First, we want to address the adaptability to different diseases. We made several changes. We now write:
   In the end, model (1) can be substituted with any type of (complex) ODE-based model (not yet integrating mobility processes). However, the modeling of the transmission process can be very different. In other models, a single parameter, often referred to as β, is used to represent the transmission dynamics [1, 2, 3] and [4, 5, 6, 7] directly integrate transmission on or after commuting in the force of infection. In the given presentation, we split this parameter into two different factors, the transmission probability per contact ρ and the contact rate ϕ. This decomposition allows for a more detailed and flexible parametrization of the transmission process. On top of this splitting, we add a second model realizing transmission on commuting and travel. This additional model can be used, independent of the underlying transmission dynamics model. We admit that by this splitting, seasonal mobility effects are not yet taken into account. However, seasonality is integrated by a parametric trigonometric curve as first described in [8]. This means that seasonality can also be interpreted as a change in the effective contact rate over time. For more details also see (16).

   With regard to seasonality and significantly changed travel activity during vacation periods, such as the summer vacations, it can be said that our model already deals with several of these aspects. First of all, the given geometry induces that mobility is mainly driven by adults as schools are often in the same county. Lower to no contacts of pupils (in schools) are realized through so called dampings of the (school) contact matrix as also already described in [8]. We however agree that vacation mobility, in particular with large-distance travel is difficult to reliably meet. We admit that our model is not (yet) adapted for, e.g., computing the import risk from hot spots outside Germany and that additional models (and data) might be needed for vacation time. This however is not the main focus of our improvement and

could be addressed with another model such as [9].
We now write:
Especially for the school contact location, vacation times are important. We have thus implemented the federal state dependent vacation times by adjusting the (school) contact matrix in the corresponding periods. We admit that our model is not (yet) adapted for, e.g., computing the import risk from hot spots outside Germany and that additional models (and data) might be needed for vacation time. This however is not the main focus of our improvement and could be addressed with other established models such as [9]. On average, however, as reported in previous publications [10, 8], the parametric trigonometric curve implemented for seasonality fits very well the observed surges and declines of infections over the year.
and
Furthermore, $\rho_{M,i}^{(k)}$ is the transmission risk per contact which is modeled through a baseline transmission risk $\rho_{0,N,i}^{(k)}$, a potential reduction factor for better-protected individuals $p_{E,PI}$ and $p_{E,II}$ and a parametric trignometric curve

$$\rho_{M,i}^{(k)} = \rho_{0,M,i}^{(k)} \left(1 + k \sin\left(\pi\left(\frac{t}{182.5} + \frac{1}{2}\right)\right)\right), \tag{1}$$

with $\rho_{0,PI,i}^{(k)} = p_{E,PI}\, \rho_{0,N,i}^{(k)}$ and $\rho_{0,II,i}^{(k)} = p_{E,II}\, \rho_{0,N,i}^{(k)}$ and where t is the day of the year.

2. *The authors assume that the number of contacts in non-traffic locations is independent of the additional contacts during travel. The authors have to justify such a strong assumption.*

   Answer: We agree with the reviewer that no strong assumption should go without justification. We might have incorrectly formulated this assumption. What we basically meant was that the number of contacts at home, workplaces, schools or leisure does not get reduced if a person makes more or less contacts in public transport/on commuting. Although we see that the time spent in travel mode reduces the total available time on a day, the sole fact of using public transport does not lead to the assumption that a person meets a smaller number of friends, colleagues or family members (on average). By disaggregating the POLYMOD contact data, we assume that both, subpopulations, intercounty commuters and others, have the same average number of contacts in home, work, or school places. We now write:
   We further assume that the number of contacts in nontraffic locations, $\phi_{nt}^{(k)}(t)$, is independent of the additional contacts during travel, e.g., the sole fact that a person commutes or uses public transport does (on average) not reduce the number of contacts to family members, friends, or colleagues.

3. *Given that the interest is in disease dynamics across Germany, the authors neglect the importation risk from different areas outside of Germany. Please comment on this.*

Answer: As also mentioned before, this is a limitation of our model realization presented here. Let us note that the model could be extended to also couple countries or regions outside Germany. This would only need reliable data on, e.g., intercounty mobility – which is difficult to assess for all connected regions (abroad) or foreign countries. A suitable model for future connection could be given by, e.g., [9]. However, this limitation is of minor importance with lower influence if we do not consider time spans where a) the import of a new variant of concern has to be considered or if b) abroad regions exhibit substantially larger incidences such that the factor "abroad-mobility * incidence-difference" become significant.
We added the corresponding sentence to the limitations:
Furthermore, we have focused on daily mobility activities in this study. The seasonal impact of, e.g., vacation times on travel behavior were not taken into account and influences from abroad have not been included here. For the summer vacation time, where travel activities significantly change with travel from hot or to hot spot regions (outside Germany), our model might need enrichment by an import risk model to reliably capture disease dynamics from the outside.

4. *The model is an SEIR-type model with immunity waning. I suggest to replace SECIRS by SEIRS to avoid any misleading to the reader.*

   Answer: We feel that there might have been some confusion. With the given notation we adhere to the introduction of the model introduced in [11] where the letter C was explicitly used to denote pre- or asymptomatic transmission after a noninfectious exposure period – which is also modeled similar in the paper submitted here. We also refer to item 9 below and would like to note that also other authors use the C notation explicitly, see, e.g., [12, 13, 14].

5. *The model lacks of the literature on immunity waning. It is important that the authors discuss how immunity wanes with respect to SARS-CoV-2 but also including latest research on modes of waning.*

   Answer: We thank the reviewer for highlighting an incomplete view on the literature on immunity waning for SARS-CoV-2. Although, we admit that we can probably not cover all articles, we have significantly extended this section, in particular based on two systematic reviews and meta-analyses. We now write:
   To motivate and, later, parametrize our model, we first looked into [15], which had considered Influenza with vaccination and reinfections (with new variants) as well as an early result for SARS-CoV-2 [16]. We then also considered two recent systematic reviews with 26 studies in [17] and 65 studies from 19 different countries in [18]. According [17], protection against hospitalisation or serious illness due to previous infections was found to be 74.6% (on average) after 12 months, waned from 80-90 % after two months. In individuals with hybrid immunity, i.e., a combination of infection and vaccination, the protective effect of hybrid

immunity following primary series remained high at 97.4% after 12 months and 95.3% after 6 months following the first booster vaccination – with practically no waning detected. The paper also shows that protection against reinfection after a previous SARS-CoV-2 infection decreases from roughly 70 % after two months to 24.7% within 12 months. For individuals with hybrid immunity with primary series vaccination, protection against reinfection after the first booster vaccination fell from 75-80 % to 41.8% within one year interval. Individuals with hybrid immunity due to first booster vaccination had a protection of 46.5% after 6 months. A further systematic review and meta-analysis by [18] found similar results. Protection against (any) reinfection by the Omicron BA.1 variant fell to around 36.1% after 40 weeks, with slightly faster decline against symptomatic reinfection. Protection remained higher for earlier variants but also decreased over time. Again, protection against serious illness stayed high for all variants, including Omicron BA.1, i.e., at 88.9% after 40 weeks. Earlier variants even showed protection of over 90%. These results confirm that despite the decline in protection against reinfection, protection against severe courses lasts longer and remains robust. Overall, we conclude that the immunity and decline in immunity plays a major role in the course of time and should be considered with two different paces.
and Given the literature findings on waning immunity as mentioned in the introduction of the waning immunity model, we implement a rather quick immune waning for any transmission, over several months, and use $T_{\mathcal{I}_{PI}} = T_{\mathcal{I}_{II}} = 60$ days. To ensure a long-term protection against severe infection, we set $T_{W_{PI}} = T_{W_{II}} = 720$ days, which means that very little waning happens in a one year time period.

6. *Given the high complexity level of the proposed model, the authors need to specify in what way their model improve upon traditional models. For instance, they need to discuss how their model behave with respect to models with no immunity waning, given a few months time frame.*

Answer: We agree with the reviewer that more results on the immunity waning itself need to be presented (from which we refrained in first submission due to the already lengthy article). We have now added simulation results for an aggregated model (where we explicitly separate the mobility model from the immunity waning) on a two years time frame (2022-2024). To indicate how the new model advances prior models, we use wastewater samples from the AMELAG [19] project that more adequately capture disease dynamics in times of changing or absent testing strategies. While we see that the new model qualitatively represents declines and resurges over the two year period, several (local) peaks induced by heterogeneous spread can only be captured by an aggregated peak over a larger time frame. We also included the prior model [20] to show how it inadequately results in declining and eventually removed disease dynamics.
We now write:

Analysis of a global SECIRS-type model using wastewater data

Given the complexity of our proposed model, it is important to show the advancements on the particular elements separately. Therefore, we consider the results of the immune-waning model separately from the impact of the mobility scheme and use a single (waning immunity) model for the whole of Germany over a two years time frame (2022-2024). The parameters used in this analysis differ slightly from those used in the spatially resolved model (Table 3 and Table 1) since we homogeneously aggregate heterogeneous immunity dynamics over the whole of Germany. We prevented from tweaking parameters too much to improve the global fit, as inferred parameters would necessarily be wrong on a local scale. We use wastewater data as an unbiased estimator to adequately capture disease dynamics in times of changing or absent testing strategies. From Fig. 1, we see that the new model qualitatively represents the three major surges and declines over the two year period indicated by the wastewater samples from the AMELAG [19]. On the other hand, the old model wrongly results in an eradication of the virus in the population. We furthermore see that the development of reported cases diverges significantly from the wastewater measurements. We see that the global model does not fit well several local peaks induced by a heterogeneous spread of COVID-19 in Germany, see also the local wastewater measurements in the Appendix. Additional considerations to use and validate our novel model in different scenarios will be the focus of future work.

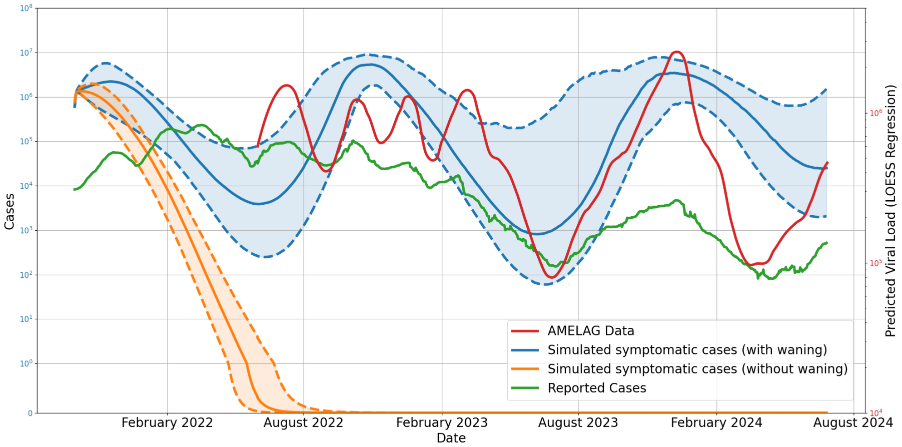

Figure 1: **Comparison of the simulated and reported cases with wastewater measurements over a two-year period (2022-2024).** New symptomatic infected individuals simulated with novel immune-waning model (blue) and existent no-waning model (orange). Obtained AMELAG wastewater data (red) and reported cases (green). The solid line represents the median of the simulation results, while the shaded area indicates the 25th to 75th percentile.

Table 1: **Model parameters for the global SECIRS-Type model without spatial resolution.** The table shows the basic transmission probability ($\rho_i^{(0)}$) and the increased protection ($p_{E_{PI}}$ and $p_{E_{II}}$) for individuals with higher immunity.

Parameter	Age Group					
	0-4	5-14	15-34	35-59	60-79	80+
$\rho_{0,N}$	$[0.06, 0.12]$	$[0.15, 0.21]$			$[0.24, 0.3]$	$[0.3, 0.4]$
$p_{E_{PI}}$	0.4					
$p_{E_{II}}$	0.4					

7. *Later, the authors compared their model with the one given in [17]. From Fig. 13, the epidemic shape shows different patterns. The robustness of this result is not addressed by the paper. Also, such a result was obtained using a fictional outbreak scenario. The authors have to clarify how these fictional data were obtained as well as how robust is their result.*

   Answer: We agree with the reviewer that more validation should have taken place. We have addressed this remark by one minor and two major changes.

   a) For the fictional outbreak scenario, we provide more details as given below.
   b) We have added a theoretical section and numerical validation that the novel model is in fact a generalization of the previous model with travel time larger zero.
   c) We have added a simulation for Post-Munich Oktoberfest until the beginning of the winter seasons in 2022 where we meet ICU admission developments over a three months time frame.

   a) For on how the fictional scenario results are obtained, we write:
   ”In this section, we want to show the impact of the mobility on the evolution of the disease using a fictional outbreak scenario. Therefore, we assume that in the initial stage to only have a single county which has a high amount of exposed people. In this context, we assume 5 % of Cologne’s population to be exposed to the virus. Although this number is immense, it was chosen by intention to directly visualize the qualitative propagation of new cases to connected counties.

   To emphasize the influence of mobility, we compared our results with the mobility scheme presented in [8] (here denoted *existing instantaneous travel method*, both times using our multi-layer waning immunity model. The advantage of the novel mobility approach accounting for travel and transmission during commuting, contrasting the immediate transfer of travelers in [8].”

b) To highlight the advantages of the proposed model, we further investigate the theoretical properties of the novel mobility scheme. We demonstrate that the novel model is also a theoretical generalization of the model presented in [17]. We show that, as travel times goes to zero, the novel approach simplifies and converges to the results of the existing instantaneous travel mobility scheme [17]. We have added a section "Theoretical properties of the novel model" in the section "Materials and Methods" as well as a section "Numerical analysis of the novel mobility scheme" to validate the theoretical findings.

Theoretical properties of the novel mobility model

In order to give the motivation for our scaling, we assume constant contact patterns over the considered day, i.e., $\phi_{nt}^{(k)}(t) = \phi_{nt}^{(k)}$ and $\phi_{tr}^{(k)}(t) = \phi_{tr}^{(k)}$, $t \in [0,1]$. For the total number of contacts, we then directly obtain

$$\begin{aligned}
&\int_0^{t_{tr}^{(k)}} N^{(k)} \left((1-p_{tr}^{(k)})\phi_{nt}^{(k)} + p_{tr}^{(k)} \frac{\phi_{nt}^{(k)}}{t_{tr}^{(k)}} \right) \mathrm{d}t + \int_{t_{tr}^{(k)}}^1 (1-p_{tr}^{(k)}) N^{(k)} \phi_{nt}^{(k)} \mathrm{d}t \\
&\qquad + \int_{t_{tr}^{(k)}}^1 p_{tr}^{(k)} N^{(k)} \frac{\phi_{tr|tr}^{(k)}}{1-t_{tr}^{(k)}} \mathrm{d}t \\
=& N^{(k)} \left((1-p_{tr}^{(k)})\phi_{nt}^{(k)} t_{tr}^{(k)} + p_{tr}^{(k)}\phi_{nt}^{(k)} + (1-p_{tr}^{(k)})\phi_{nt}^{(k)}(1-t_{tr}^{(k)}) + \phi_{tr}^{(k)} \right) \\
=& N^{(k)} \left(\phi_{nt}^{(k)} + \phi_{tr}^{(k)} \right),
\end{aligned} \tag{2}$$

where we recognize Eq (2).

Let us note that the parameters of baseline contacts in traffic $\phi_{tr}^{(k)}$ as well as the travel time $t_{tr}^{(k)}$ that implicitly appear in (7) need to fit together, e.g., coming from reliable data sources of the same country. That means that the model outcome for, e.g., fixed $\phi_{tr}^{(k)}$ and arbitrary $t_{tr}^{(k)}$ is not anymore in line with the data used in the model. However, in the following sections, we still allow a certain variance in travel time between different regions, i.e., $1 - t_{tr}^{\max} < 1 - t_{tr}^{(k)} < 1 - t_{tr}^{\min}$, $k \in \{1, \dots, n\}$. Due to data sparsity, i.e., no individual traffic contact records for arbitrary commuting routes ($\phi_{tr}^{(k)} = \phi_{tr}$, $k \in \{1, \dots, n\}$), we only assume that

$$\frac{\phi_{tr}}{1 - t_{tr}^{\max}} := \text{const} \tag{3}$$

to consider the limit case without travel times, instead of directly considering (7).

Considering the limit $t_{tr}^{(k)} \to 1$ implies $\phi_{tr}^{(k)} \to 0$ (with the same convergence rate). From (2), we have

$$\phi^{(k)}(t) \to \phi_{nt}^{(k)}(t) \tag{4}$$

and (5) also reduces to (10) on $t \in [0, 1]$. The corresponding IVPs (6) and (7) can be dropped as the interval converges to a null set and the parameters remain bounded.

Thus, we see that the novel model advances the previous model [8] by removing the unrealistic assumption of instantaneous travel and allowing for realistic travel times $1 - t_{tr}^{(k)}$.

We also added a numerical validation of these findings:

Numerical validation of novel mobility scheme

In the section "Theoretical properties of the novel mobility model", we have already shown that the novel mobility scheme reduces to the previously suggested model [8], as the travel time approaches zero. For validation, we here present the numerical convergence of the novel model to the previously proposed model. To simplify disease dynamics, we use local SIR models (1) for the five German counties Cologne, Leverkusen, Rheinisch-Bergischer Kreis, Rhein-Sieg-Kreis, and Bonn with realistic demographies and parameters as given in Fig. 2. For the initial conditions, we set 0.1% of the total populations to be infected and zero recovered individuals. We then compute the solution over 50 days using the novel mobility scheme and subsequently evaluate the difference to the solution obtained with the existing instantaneous travel mobility scheme [8] at the last date. Table 2 shows that, as travel time decreases, the difference between the two model outputs converges to zero. Additionally, we present the convergence behavior using map plots for the different values of t_{tr}^{max} in Fig. 2.

Table 2: **Numerical convergence of the novel mobility scheme.** The table shows the normed difference between the outcomes of the previous and the novel mobility scheme after 50 days of simulation with values of $t_{tr}^{max} \to 0$.

	**Difference**					
t_{tr}^{max}	*Total*	*Cologne*	*Leverkusen*	*Rhein.-Berg. Kreis*	*Rhein-Sieg-Kreis*	*Bonn*
$1.00e+00$	$1.10e+05$	$4.27e+04$	$1.18e+04$	$1.48e+04$	$2.46e+04$	$1.61e+04$
$1.00e-01$	$7.81e+03$	$3.12e+03$	$8.69e+02$	$6.12e+02$	$1.73e+03$	$1.48e+03$
$1.00e-02$	$7.58e+02$	$2.92e+02$	$9.18e+01$	$1.05e+02$	$1.69e+02$	$1.01e+02$
$1.00e-03$	$7.56e+01$	$2.91e+01$	$9.15e+00$	$1.04e+01$	$1.69e+01$	$1.01e+01$
$1.00e-04$	$7.55e+00$	$2.90e+00$	$9.14e-01$	$1.04e+00$	$1.69e+00$	$1.01e+00$
$1.00e-05$	$7.55e-01$	$2.90e-01$	$9.14e-02$	$1.04e-01$	$1.69e-01$	$1.01e-01$
$1.00e-06$	$7.55e-02$	$2.90e-02$	$9.14e-03$	$1.04e-02$	$1.69e-02$	$1.01e-02$
$1.00e-07$	$7.55e-03$	$2.90e-03$	$9.14e-04$	$1.04e-03$	$1.69e-03$	$1.01e-03$
$1.00e-08$	$7.55e-04$	$2.90e-04$	$9.14e-05$	$1.04e-04$	$1.69e-04$	$1.01e-04$
$1.00e-09$	$7.55e-05$	$2.90e-05$	$9.14e-06$	$1.04e-05$	$1.69e-05$	$1.01e-05$
$1.00e-10$	$7.55e-06$	$2.90e-06$	$9.14e-07$	$1.04e-06$	$1.69e-06$	$1.01e-06$
$1.00e-11$	$7.56e-07$	$2.91e-07$	$9.11e-08$	$1.05e-07$	$1.68e-07$	$1.01e-07$

Unfortunately, the tracing of infections to the place of transmission is a tedious and often impossible task – as long as no perfect surveillance of infections and mobility patterns (or contacts) was done. However, it is realistic to assume that transmissions happen on commuting and that perfect protection cannot be realized in public transport settings, even if, e.g., face masks are worn or other mitigation strategies are executed. With the new model we remove the unrealistic assumption that transmission on commuting itself cannot happen and that mobility is an instant process. That means that directly from a mechanistic view, the new model advances the old one.

c) Furthermore, we validated our model on Post-Munich Oktoberfest 2022 (until the beginning of the winter season) and the propgation of mild and ICU cases over a period of several months.
There, we write:

Developments of Post-Oktoberfest until winter season 2022 in Bavaria

To further validate the effect of the novel mobility model, we simulate the Post-Munich Oktoberfest period until the beginning of the winter season 2022. The Oktoberfest, which took place from September 17 to October 3, 2022 and attracted around 5.7 million visitors [21]. Unfortunately, conducted tests have decreased significantly over 2022 and

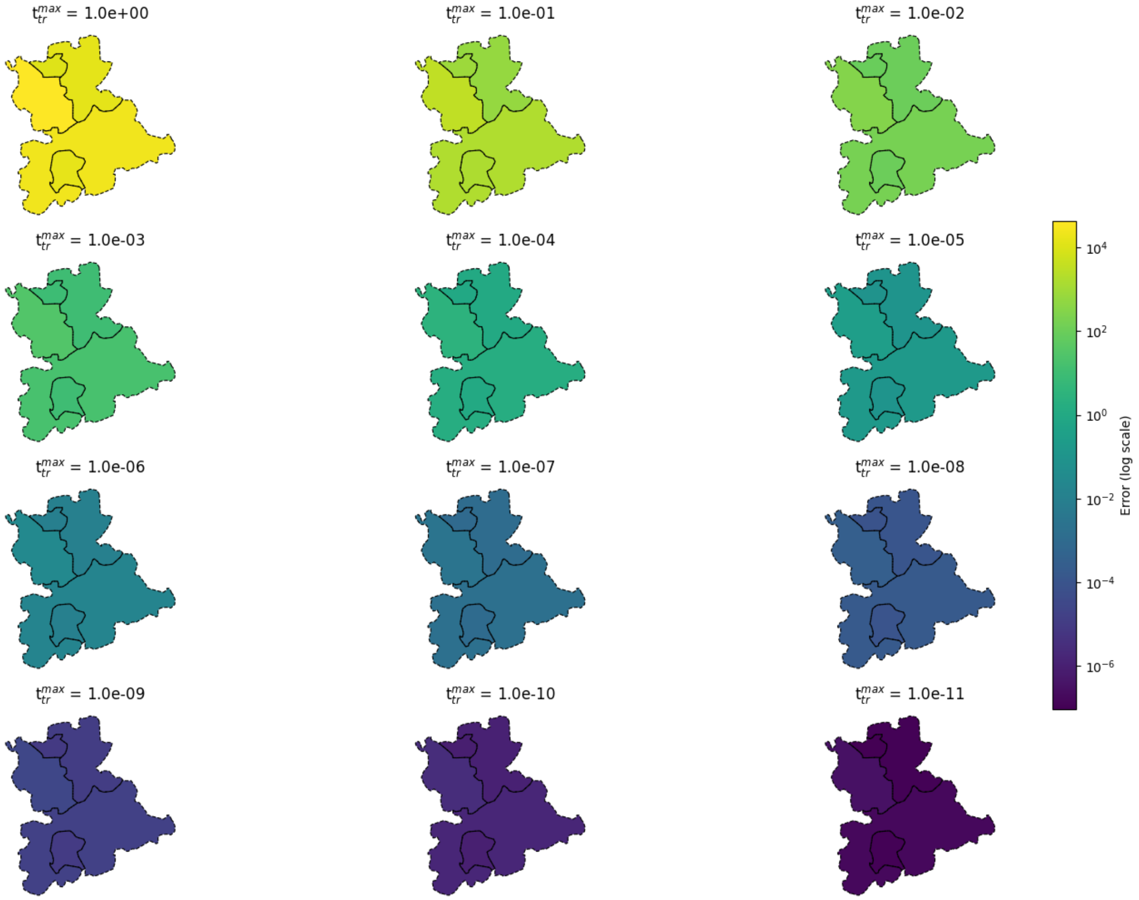

Figure 2: **Numerical convergence of the novel mobility scheme on map plots.** The figure presents a grid of maps, each illustrating the spatial distribution of normed difference as provided in Table 2 for the respective county. The counties displayed are Cologne, Leverkusen, Rheinisch-Bergischer Kreis, Rhein-Sieg-Kreis, and Bonn. The maps use a logarithmically scaled colorbar. Using geodata "Verwaltungsgebiete 1:2 500 000, Stand 01.01. (VG2500)" from https://gdz.bkg.bund.de, copyright; GeoBasis-DE / BKG 2021, license dl-de/by-2–0, see https://www.govdata.de/dl-de/by-2-0.

positive rate has been high, see [22] and Fig. 3, which makes reported case numbers quite unreliable and comparisons difficult. On an aggregated level we thus also include reported ICU numbers from [23]. We initialize the model based on the official reported data one week after the festival, at October 10 2022, and simulate until the end of the year using the parameters in Table 3. We adjusted the transmission probability ρ with a factor of 0.8. While the simulate mild cases largely exceed the reported cases (see Fig. 3 (left)),

which is no surprise given the low number of tests and high positive rate, the simulated ICU admissions come close to the reported ICU admissions; see Fig. 3 (right).

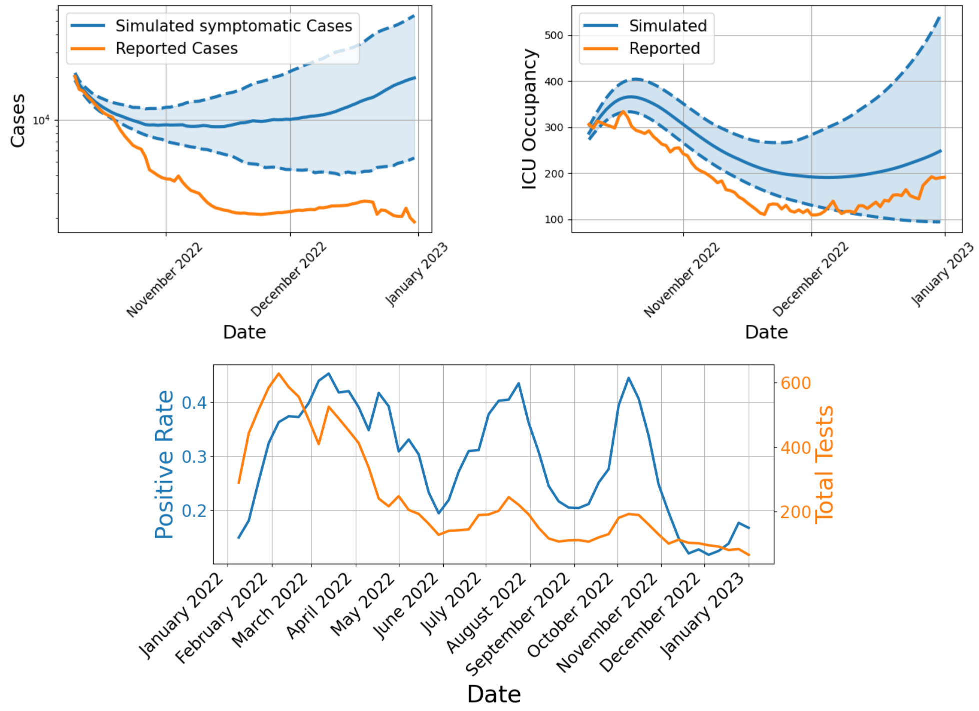

Figure 3: **Comparison of simulated and reported data for Bavaria and conducted tests.** The top left figure shows simulated daily new symptomatic against reported cases in Bavaria, with data smoothed using a 7-day moving average to reduce oscillations. The top right figure compares simulated ICU admissions to reported ICU data. The bottom figure presents the total number of tests conducted alongside with the positive test rate in Bavaria for the entire year 2022.

To provide spatial details of our simulations, we present the simulated number of symptomatic infections in a spatially resolved plot of Bavaria. In Fig. 4, we see the spread of infections across the federal state, with the numbers presented relative to the local population.

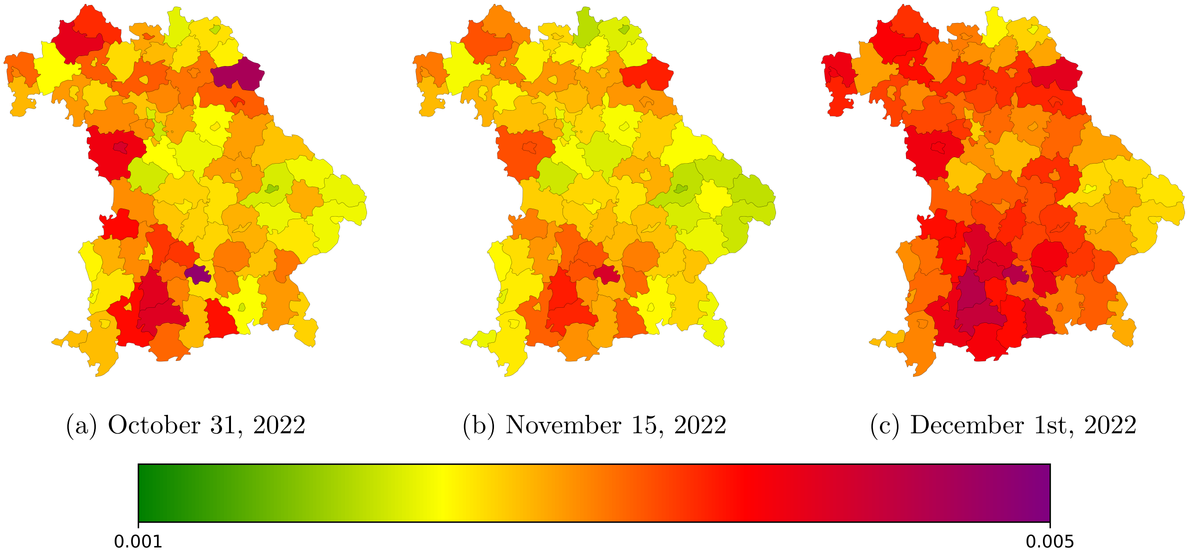

Figure 4: **Spatial distribution of symptomatic infections in Bavaria for selected days of the Post-Oktoberfest and beginning winter season 2022.** Presented values are provided relative to local population.

8. *As the model used is highly parameterized, a sensitivity analysis is needed to identify the parameters that have the most impact on the disease dynamics.*

Answer: We have also addressed this point and now provide a sensitivity analysis of the immunity-waning-model with respect to new infections and ICU occupancy in the appendix. We now write:
For future developements and in order to determine the most influential parameters with respect to different the model outcomes, we performed a sensitivity analysis using the Morris screening method [24]. This method calculates elementary effects for each parameter by systematically varying one parameter at a time while leaving other parameters unchanged. We present the elementary effects of the individual parameters with respect to new infections and number of deaths in the appendix and Fig. 19

In short, the sensitivity analysis indicates that the transmission risk, followed by the protection factors against any or symptomatic infection, the reduction of the infectious time for protected individuals and the overall infectious times have the highest influence on the number of new infections. The high influence of the transmission risk suggests that small changes in the probability of transmission upon contact can lead to significant variations in

number of daily new infections. It should also be mentioned that a change in the probability of infection due to commutativity is equivalent to a change in the contact rate and therefore the result also applies to the latter. In terms of ICU occupancy, the analysis indicates that the probabilities of transition from the symptomatic to the severe state and from the severe state to the critical state have the highest impact, shortly followed by the transmission risk. For more details, we refer to Fig. 19 in the appendix.

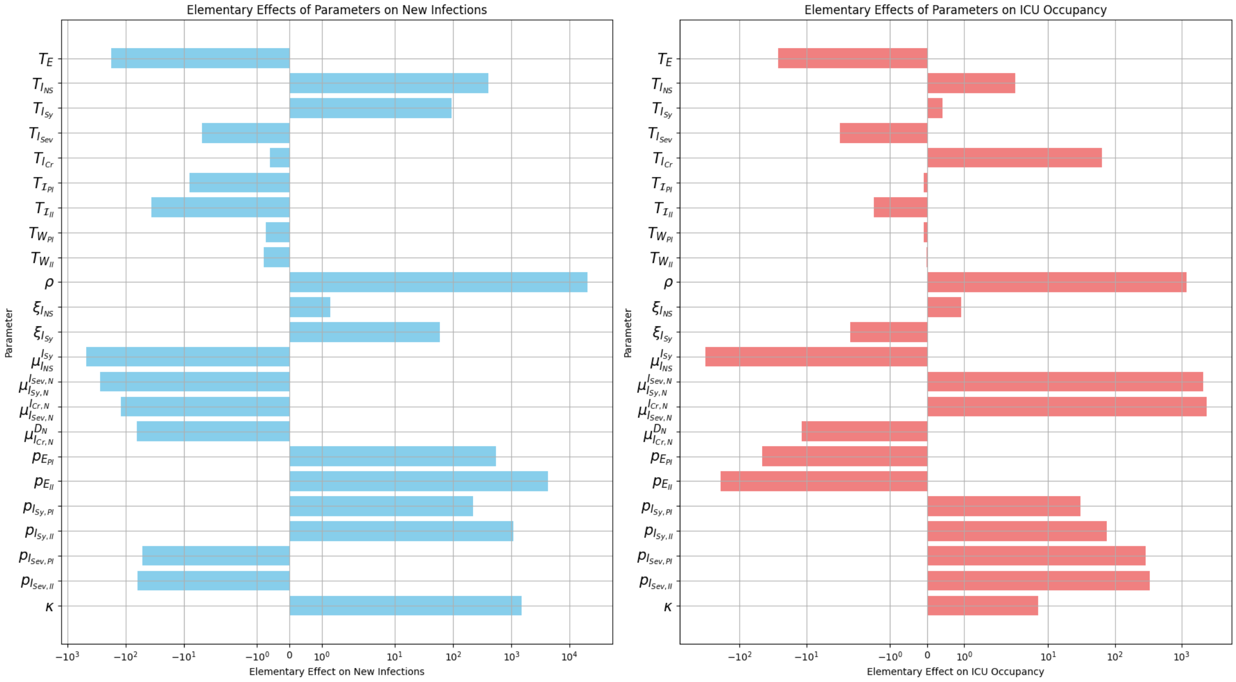

Figure 5: Results of the sensitivity analysis showing the Elementary Effects for the model parameters with respect to the daily new infections (left) and ICU occupancy (right). The impact of each parameter is represented by a logarithmic scaled bar where the length represents the positive/negative influence.

9. *It is known that a large portion of transmissions happen before symptoms onset. The introduction of a presymptomatic compartment would be of great interest.*

   Answer: We perfectly agree with a reviewer and would like to highlight that this has already been the case with the first submission. We have slightly adapted our explanations to make this more prominent.
   We now write:
   First, as pre- or asymptomatic transmission plays an important role for SARS-CoV-2, we use

a differentiation in nonsymptomatic and symptomatic transmission to separate their related contributions to the disease dynamics.

10. *Some new parameters appear in Table 3:* $p_{H_{PI}}$ *and* p_{H_v}*! What are those parameters? I couldn't see them in the model definition.*

    Answer: We thank the reviewer for the very attentive reading and spotting this typo which results from a notation we used previously. We have corrected these parameters to be $p_{I_{Sev,PI}}$ and $p_{I_{Sev,II}}$.

11. *The authors claim that the parameter values used for Omicron and in Table 3 are justified from the literature. The authors said to refer to [34,35], however, I couldn't find where the values of the exposure and infectious periods of* $\approx$ *0.6 come from.*

    Answer: We thank the author for spotting this incorrect presentation. Instead of the parameters, we accidentally have printed the value of 1/parameter.
    Let us also directly explain how the correct values have been obtained as the determination of these values might not be straightforward. We have relied on the work and assumptions of [11, 8] to derive these parameters. We added the first paper to the cited column in the table. There, the values for TimeExposed and TimeInfectedNoSymptoms are obtained using the serial interval and the incubation time. The references we have cited provide the values for the serial interval and incubation time. To clarify, based on the cited literature, we used a serial interval (SI) of 2.38 days and an incubation period (INC) of 3.1 days. We calculated the given transition times using the method described in [11, 8].

$$T_E = 2SI - INC = 1.66$$
$$T_{I_{NS}} = 2(INC - SI) = 1.44$$

Minor comments:

1. *Legend in Fig 2. show* $\phi = 9.1$ *and* $\phi = 9.05$*. Please correct these legends.*
   Answer: We thank the reviewer for attentive reading and for spotting the error in the figures. We have corrected this. The value for ϕ is indeed 9.1 as it results from eq. (5) in the submitted version. The figures have been corrected.

2. *Line 184. j and k should be written in mathematical style. Check the whole manuscript*
   Answer: We thank the reviewer for attentive reading. This has been corrected.

3. *Line 208: An important*
   Answer: We thank the reviewer for attentive reading. This has been corrected.

4. *Line 432: is number*
   Answer: We thank the reviewer for attentive reading. This has been corrected.

References

[1] Brauer F, Castillo-Chavez C, Feng Z. Mathematical models in epidemiology. vol. 32. Springer; 2019.

[2] Marinov TT, Marinova RS. Adaptive SIR model with vaccination: simultaneous identification of rates and functions illustrated with COVID-19. Scientific Reports. 2022;12(1). doi:10.1038/s41598-022-20276-7.

[3] Peng R. Asymptotic profiles of the positive steady state for an SIS epidemic reaction–diffusion model. Part I. Journal of Differential Equations. 2009;247(4):1096–1119. doi:https://doi.org/10.1016/j.jde.2009.05.002.

[4] Pei S, Kandula S, Shaman J. Differential effects of intervention timing on COVID-19 spread in the United States. Science Advances. 2020;6(49):eabd6370. doi:10.1126/sciadv.abd6370.

[5] Chen X, Zhang A, Wang H, Gallaher A, Zhu X. Compliance and containment in social distancing: mathematical modeling of COVID-19 across townships. International Journal of Geographical Information Science. 2021;35(3):446–465. doi:10.1080/13658816.2021.1873999.

[6] Levin MW, Shang M, Stern R. Effects of short-term travel on COVID-19 spread: A novel SEIR model and case study in Minnesota. PLOS ONE. 2021;16(1):e0245919. doi:10.1371/journal.pone.0245919.

[7] Liu J, Ong GP, Pang VJ. Modelling effectiveness of COVID-19 pandemic control policies using an Area-based SEIR model with consideration of infection during interzonal travel. Transportation Research Part A: Policy and Practice. 2022;161:25–47. doi:10.1016/j.tra.2022.05.003.

[8] Kühn MJ, Abele D, Mitra T, Koslow W, Abedi M, Rack K, et al. Assessment of effective mitigation and prediction of the spread of SARS-CoV-2 in Germany using demographic information and spatial resolution. Mathematical Biosciences. 2021; p. 108648. doi:https://doi.org/10.1016/j.mbs.2021.108648.

[9] Klamser PP, Zachariae A, Maier BF, Baranov O, Jongen C, Schlosser F, et al. Inferring country-specific import risk of diseases from the world air transportation network. PLOS Computational Biology. 2024;20(1):e1011775. doi:10.1371/journal.pcbi.1011775.

[10] Bauer S, Contreras S, Dehning J, Linden M, Iftekhar E, Mohr SB, et al. Relaxing restrictions at the pace of vaccination increases freedom and guards against further COVID-19 waves. PLOS Computational Biology. 2021;17(9):e1009288. doi:10.1371/journal.pcbi.1009288.

[11] Khailaie S, Mitra T, Bandyopadhyay A, Schips M, Mascheroni P, Vanella P, et al. Development of the reproduction number from coronavirus SARS-CoV-2 case data in Germany and implications for political measures. BMC medicine. 2021;19(1):1–16.

[12] Mann J, Roberts M. Modelling the epidemiology of hepatitis B in New Zealand. Journal of Theoretical Biology. 2011;269(1):266–272. doi:https://doi.org/10.1016/j.jtbi.2010.10.028.

[13] Gottwald T, Poole G, McCollum T, Hall D, Hartung J, Bai J, et al. Canine olfactory detection of a vectored phytobacterial pathogen, Liberibacter asiaticus, and integration with disease control. Proceedings of the National Academy of Sciences. 2020;117(7):3492–3501.

[14] Dorn F, Khailaie S, Stoeckli M, Binder SC, Mitra T, Lange B, et al. The common interests of health protection and the economy: evidence from scenario calculations of COVID-19 containment policies. The European Journal of Health Economics. 2023;24(1):67–74.

[15] Patel MM, York IA, Monto AS, Thompson MG, Fry AM. Immune-mediated attenuation of influenza illness after infection: opportunities and challenges. The Lancet Microbe. 2021;2(12):e715–e725. doi:10.1016/s2666-5247(21)00180-4.

[16] Nordström P, Ballin M, Nordström A. Risk of infection, hospitalisation, and death up to 9 months after a second dose of COVID-19 vaccine: a retrospective, total population cohort study in Sweden. The Lancet. 2022;399(10327):814–823. doi:10.1016/S0140-6736(22)00089-7.

[17] Bobrovitz N, Ware H, Ma X, Li Z, Hosseini R, Cao C, et al. Protective effectiveness of previous SARS-CoV-2 infection and hybrid immunity against the omicron variant and severe disease: a systematic review and meta-regression. The Lancet Infectious Diseases. 2023;doi:https://doi.org/10.1016/S1473-3099(22)00801-5.

[18] Stein C, Nassereldine H, Sorensen RJD, Amlag JO, Bisignano C, Byrne S, et al. Past SARS-CoV-2 infection protection against re-infection: a systematic review and meta-analysis. The Lancet. 2023;401(10379):833–842. doi:10.1016/s0140-6736(22)02465-5.

[19] Robert Koch-Institut F. Abwassersurveillance AMELAG; 2024. Available from: https://doi.org/10.5281/zenodo.13683260.

[20] Koslow W, Kühn MJ, Binder S, Klitz M, Abele D, Basermann A, et al. Appropriate relaxation of non-pharmaceutical interventions minimizes the risk of a resurgence in SARS-CoV-2 infections in spite of the Delta variant. PLOS Computational Biology. 2022;18(5):e1010054. doi:10.1371/journal.pcbi.1010054.

[21] Statista. Anzahl der Besucher auf dem Oktoberfest von 1980 bis 2023; 2024. Available from: https://de.statista.com/statistik/daten/studie/165511/umfrage/anzahl-der-besucher-auf-dem-oktoberfest-seit-1980/.

[22] Bayerisches Landesamt für Gesundheit und Lebensmittelsicherheit. Anzahl der Laboruntersuchungen auf SARS-CoV-2 in Bayern (Tabelle 11); 2023. Available from: https://www.lgl.bayern.de/gesundheit/infektionsschutz/infektionskrankheiten_a_z/coronavirus/karte_coronavirus/archiv2.htm.

[23] Deutsche Interdisziplinäre Vereinigung für Intensiv- und Notfallmedizin (DIVI). DIVI Intensivregister Tagesreport; 2022. Available from: https://www.divi.de/divi-intensivregister-tagesreport-archiv.

[24] Morris MD. Factorial Sampling Plans for Preliminary Computational Experiments. Technometrics. 1991;33(2):161–174. doi:10.1080/00401706.1991.10484804.

Reviewer #2

The authors present a new epidemiological model that introduces novelty in two fronts: a/ travel time in a graph-ODE metapop model (including passing explicitly through multiple districts), b/ a multi-layer waning immunity model that support different paces for protection against various degrees of disease severity. The authors combine these advancements to investigate the impact of mobility (more specifically the use of mitigation measures such as masks during mobility) in a late-phase epidemic scenario.

Overall, I believe the authors present interesting modelling frameworks, that cover important aspects related to the modelling of infectious diseases. I studied the proposed frameworks which appears to be sound, and I could not find any immediate errors or mistakes.

Answer: We thank the reviewer for the effort of a detailed review of our paper. We have addressed all the points mentioned by the reviewer and provide detailed answers in the following. Changes in the manuscript are highlighted accordingly.

- *However, such a complex model requires many choices, both regarding model structures and the selected parameters. I think it would be appropriate to discuss the limitations and uncertainties regarding these choices properly in the discussion section, which currently seems to be quite limited.*

  Answer: We agree with the reviewer that we had to add a few more limitations in the discussion section.
  We now write:

  The results presented in this study are based on a mean-value model, a multitude of assumptions and different parameters. Although based on an extensive literature review and previous findings in [1, 2, 3], there is still uncertainty about many (virus-specific) parameters. Although the distinction into 3-4 different immunity layers through seropositivity studies [4, 5] has been made, a division into three different subpopulations is a rough aggregation. Furthermore, our model is not suited for simulation the evolution of virus variants or the competing circulation of different variants. An inherent limitation of almost all mean-field models and most contact surveys is the difficulty to address (potential) transmission through aerosols which cannot (or only to limited extent) be measured in (direct) contacts so that estimated parameters have to address for this limitation. However, mathematical models can help to explore the mechanisms in infectious disease spread and reasonable assumptions are necessary, even for models up to digital twins. We have waived a lot of unrealistic assumptions such as homogeneous mixture in space and in demography. Our model has been resolved for meaningful regions and demography has been taken into account to model different vulnerabilities for different

age groups. In order to better assess model performance under changing parameters, we have included a sensitivity analysis on the parameters used in the waning immunity model in the appendix. The provided quantification for the different parameter effects will guide extending or reducing the model in future works and analyses.

As we stated ourselves, a critical factor in infectious disease modeling is mobility. Since the ground truth for daily mobility is difficult to grasp, again, assumptions have to be used. We have used the output of a state-of-the-art macroscopic mobility model to be used in our simulations. Different mobility patterns clearly lead to different virus distribution patterns. Furthermore, we have focused on daily mobility activities in this study. The seasonal impact of, e.g., vacation times on travel behavior were not taken into account and influences from abroad have not been included here. For the summer vacation time, where travel activities significantly change with travel from hot or to hot spot regions (outside Germany), our model might need enrichment by an import risk model to reliably capture disease dynamics from the outside.

A precise quantification of waning immunity against different courses of the disease given particular immunization histories is still an open research question. To best cope with this problem, we have considered two large and recent review articles from which we derived most reasonable parameters. However, there is still large uncertainty in several positions which we cannot reduce. Qualitatively, our model could correctly predict stagnation, decline and resurges in considered settings. The effect of waning immunity to SARS-CoV-2 over more than 1 or 2 years, the interplay with new variants or vaccinations is still an open research question our model cannot answer yet.

In order to get meaningful predictions for future developements, our model needs to initialized with reasonable values of the population's immunity. As the reporting of vaccinations is not conducted on personal level and as the dark figure of unreported transmission numbers increased with the end of the pandemic, the current status of the population is difficult to obtain. Serological studies may be needed to provide approximative starting values for future predictions. These limitations in data and assumptions should be kept in mind when interpreting the results of this work.

Additionally, to further address uncertainties, we have added a sensitivity analyses that examines the impact of different parameter of the introduced SECIRS-type model. We now write:

For future developements and in order to determine the most influential parameters with respect to different the model outcomes, we performed a sensitivity analysis using the Morris screening method [6]. This method calculates elementary effects for each parameter by systematically varying one parameter at a time while leaving other parameters unchanged.

We present the elementary effects of the individual parameters with respect to new infections and number of deaths in the appendix and Fig. 19.

In short, the sensitivity analysis indicates that the transmission risk, followed by the protection factors against any or symptomatic infection, the reduction of the infectious time for protected individuals and the overall infectious times have the highest influence on the number of new infections. The high influence of the transmission risk suggests that small changes in the probability of transmission upon contact can lead to significant variations in number of daily new infections. It should also be mentioned that a change in the probability of infection due to commutativity is equivalent to a change in the contact rate and therefore the result also applies to the latter. In terms of ICU occupancy, the analysis indicates that the probabilities of transition from the symptomatic to the severe state and from the severe state to the critical state have the highest impact, shortly followed by the transmission risk. For more details, we refer to Fig. 19 in the appendix.

- *The authors present important advances regarding metapop modelling (time-aware and passing through multiple districts) and immunity waning. However, while these components are not necessarily connected, they are evaluated in combination, which makes it hard to assess how these components match their individual goals. I would expect an evaluation on the SECIRS compartment model and an evaluation that compares the graph-ODE model from [Kuhn, 2021] with the methodological extensions presented in this paper (so, without the SECIRS model). In my opinion the comparison between the graph-ODE and the method presented in this paper can be performed on an early-phase pandemic scenario, when waning is not a concern.*

Answer: We thank the reviewer for the valuable feedback. In response to this comment, we have added three additional sections and validations. First, we present a theoretical consideration of the novel mobility model for a simple ODE-SIR-model but where basically any model (early- or late-phase) can be integrated. We show that this model converges to the already existing one in [Kuhn, 2021] if the travel time goes to zero. For this theoretical consideration, we also present a numerical validation on five randomly selected German counties. Second, we consider a global immune-waning model for Germany, showing that it can qualitatively represent overall surges and declines as observed in wastewater data. Finally, we have considered the aftermath of the Munich Oktoberfest after which ICU admissions increased in Bavaria, before shortly going down and then up again with the winter season.

In detail:

a) To highlight the advantages of the proposed model, we investigated the theoretical properties of the novel mobility scheme. We demonstrate that the novel model is also a theoretical generalization of the model presented in [17]. We show that, as travel times

goes to zero, the novel approach simplifies and converges to the results of the existing instantaneous travel mobility scheme [17]. We have added a section "Theoretical properties of the novel model" in the section "Materials and Methods" as well as a section "Numerical analysis of the novel mobility scheme" to validate the theoretical findings.

Theoretical properties of the novel mobility model

In order to give the motivation for our scaling, we assume constant contact patterns over the considered day, i.e., $\phi_{nt}^{(k)}(t) = \phi_{nt}^{(k)}$ and $\phi_{tr}^{(k)}(t) = \phi_{tr}^{(k)}$, $t \in [0,1]$. For the total number of contacts, we then directly obtain

$$\begin{aligned}
&\int_0^{t_{tr}^{(k)}} N^{(k)} \left((1-p_{tr}^{(k)})\phi_{nt}^{(k)} + p_{tr}^{(k)} \frac{\phi_{nt}^{(k)}}{t_{tr}^{(k)}} \right) \mathrm{d}t + \int_{t_{tr}^{(k)}}^1 (1-p_{tr}^{(k)}) N^{(k)} \phi_{nt}^{(k)} \mathrm{d}t \\
&\qquad + \int_{t_{tr}^{(k)}}^1 p_{tr}^{(k)} N^{(k)} \frac{\phi_{tr|tr}^{(k)}}{1-t_{tr}^{(k)}} \mathrm{d}t \qquad (1)\\
=&N^{(k)} \left((1-p_{tr}^{(k)})\phi_{nt}^{(k)} t_{tr}^{(k)} + p_{tr}^{(k)}\phi_{nt}^{(k)} + (1-p_{tr}^{(k)})\phi_{nt}^{(k)}(1-t_{tr}^{(k)}) + \phi_{tr}^{(k)} \right) \\
=&N^{(k)} \left(\phi_{nt}^{(k)} + \phi_{tr}^{(k)} \right),
\end{aligned}$$

where we recognize Eq (2).

Let us note that the parameters of baseline contacts in traffic $\phi_{tr}^{(k)}$ as well as the travel time $t_{tr}^{(k)}$ that implicitly appear in (7) need to fit together, e.g., coming from reliable data sources of the same country. That means that the model outcome for, e.g., fixed $\phi_{tr}^{(k)}$ and arbitrary $t_{tr}^{(k)}$ is not anymore in line with the data used in the model. However, in the following sections, we still allow a certain variance in travel time between different regions, i.e., $1 - t_{tr}^{\max} < 1 - t_{tr}^{(k)} < 1 - t_{tr}^{\min}$, $k \in \{1,\dots,n\}$. Due to data sparsity, i.e., no individual traffic contact records for arbitrary commuting routes ($\phi_{tr}^{(k)} = \phi_{tr}$, $k \in \{1,\dots,n\}$), we only assume that

$$\frac{\phi_{tr}}{1 - t_{tr}^{\max}} := \text{const} \qquad (2)$$

to consider the limit case without travel times, instead of directly considering (7).

Considering the limit $t_{tr}^{(k)} \to 1$ implies $\phi_{tr}^{(k)} \to 0$ (with the same convergence rate). From (2), we have

$$\phi^{(k)}(t) \to \phi_{nt}^{(k)}(t) \qquad (3)$$

and (5) also reduces to (10) on $t \in [0,1]$. The corresponding IVPs (6) and (7) can be dropped as the interval converges to a null set and the parameters remain bounded.

Table 1: **Numerical convergence of the novel mobility scheme.** The table shows the normed difference between the outcomes of the previous and the novel mobility scheme after 50 days of simulation with values of $t_{tr}^{max} \to 0$.

	**Difference**					
t_{tr}^{max}	*Total*	*Cologne*	*Leverkusen*	*Rhein.-Berg. Kreis*	*Rhein-Sieg-Kreis*	*Bonn*
$1.00e+00$	$1.10e+05$	$4.27e+04$	$1.18e+04$	$1.48e+04$	$2.46e+04$	$1.61e+04$
$1.00e-01$	$7.81e+03$	$3.12e+03$	$8.69e+02$	$6.12e+02$	$1.73e+03$	$1.48e+03$
$1.00e-02$	$7.58e+02$	$2.92e+02$	$9.18e+01$	$1.05e+02$	$1.69e+02$	$1.01e+02$
$1.00e-03$	$7.56e+01$	$2.91e+01$	$9.15e+00$	$1.04e+01$	$1.69e+01$	$1.01e+01$
$1.00e-04$	$7.55e+00$	$2.90e+00$	$9.14e-01$	$1.04e+00$	$1.69e+00$	$1.01e+00$
$1.00e-05$	$7.55e-01$	$2.90e-01$	$9.14e-02$	$1.04e-01$	$1.69e-01$	$1.01e-01$
$1.00e-06$	$7.55e-02$	$2.90e-02$	$9.14e-03$	$1.04e-02$	$1.69e-02$	$1.01e-02$
$1.00e-07$	$7.55e-03$	$2.90e-03$	$9.14e-04$	$1.04e-03$	$1.69e-03$	$1.01e-03$
$1.00e-08$	$7.55e-04$	$2.90e-04$	$9.14e-05$	$1.04e-04$	$1.69e-04$	$1.01e-04$
$1.00e-09$	$7.55e-05$	$2.90e-05$	$9.14e-06$	$1.04e-05$	$1.69e-05$	$1.01e-05$
$1.00e-10$	$7.55e-06$	$2.90e-06$	$9.14e-07$	$1.04e-06$	$1.69e-06$	$1.01e-06$
$1.00e-11$	$7.56e-07$	$2.91e-07$	$9.11e-08$	$1.05e-07$	$1.68e-07$	$1.01e-07$

Thus, we see that the novel model advances the previous model [1] by removing the unrealistic assumption of instantaneous travel and allowing for realistic travel times $1 - t_{tr}^{(k)}$.

We also added a numerical validation of these findings:

Numerical validation of novel mobility scheme

In the section "Theoretical properties of the novel mobility model", we have already shown that the novel mobility scheme reduces to the previously suggested model [1], as the travel time approaches zero. For validation, we here present the numerical convergence of the novel model to the previously proposed model. To simplify disease dynamics, we use local SIR models (1) for the five German counties Cologne, Leverkusen, Rheinisch-Bergischer Kreis, Rhein-Sieg-Kreis, and Bonn with realistic demographies and parameters as given in Fig. 2. For the initial conditions, we set 0.1% of the total populations to be infected and zero recovered individuals. We then compute the solution over 50 days using the novel mobility scheme and subsequently evaluate the difference to the solution obtained with the existing instantaneous travel mobility scheme [1] at the last date. Table 1 shows that, as travel time decreases, the difference between the two model outputs converges to zero. Additionally, we present the convergence behavior using map plots for the different values of t_{tr}^{max} in Fig. 1.

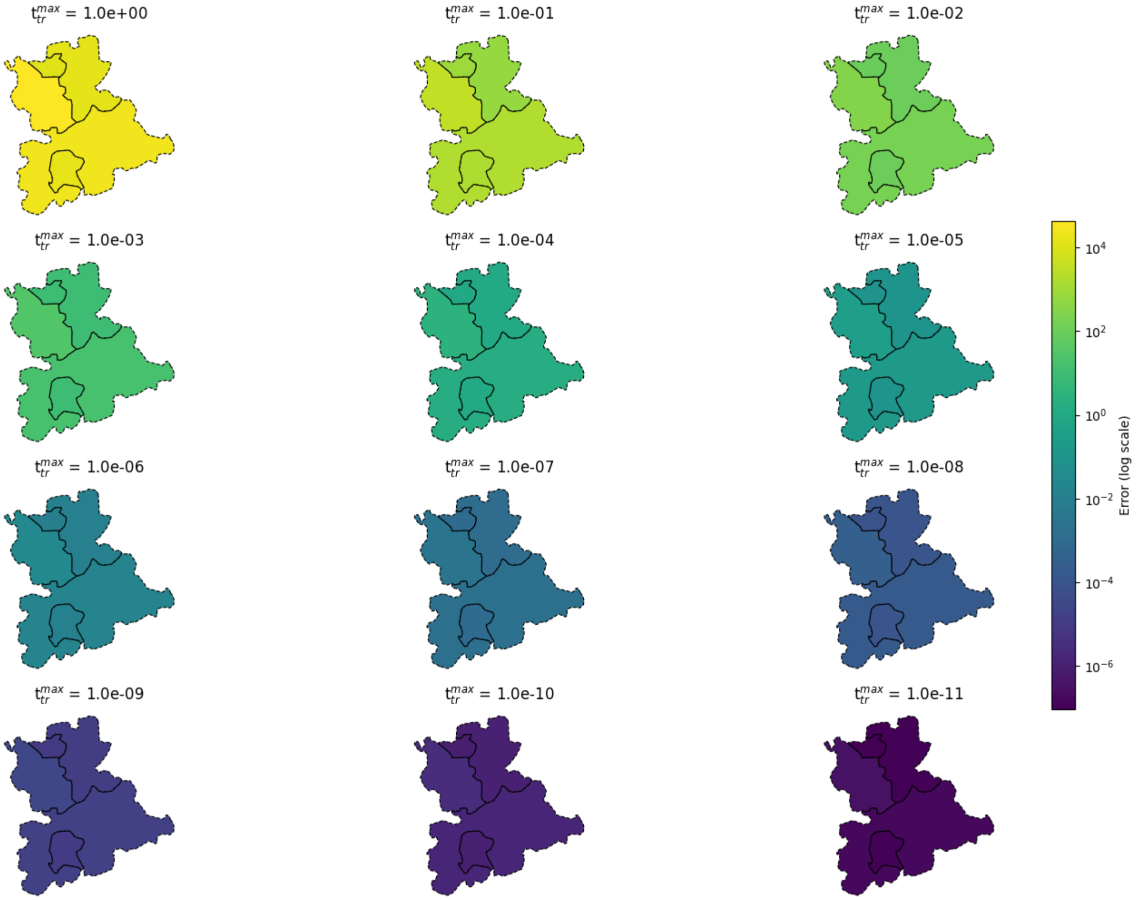

Figure 1: **Numerical convergence of the novel mobility scheme on map plots.** The figure presents a grid of maps, each illustrating the spatial distribution of normed difference as provided in Table 1 for the respective county. The counties displayed are Cologne, Leverkusen, Rheinisch-Bergischer Kreis, Rhein-Sieg-Kreis, and Bonn. The maps use a logarithmically scaled colorbar. Using geodata "Verwaltungsgebiete 1:2 500 000, Stand 01.01. (VG2500)" from https://gdz.bkg.bund.de, copyright; GeoBasis-DE / BKG 2021, license dl-de/by-2–0, see https://www.govdata.de/dl-de/by-2-0.

Unfortunately, the tracing of infections to the place of transmission is a tedious and often impossible task – as long as no perfect surveillance of infections and mobility patterns (or contacts) as done. However, it is realistic to assume that transmissions happen on commuting and that perfect protection cannot be realized in public transport settings, even if, e.g., face masks are worn or other mitigation strategies are executed. With the new model we remove the unrealistic assumption that transmission on commuting itself

cannot happen and that mobility is an instant process. That means that directly from a mechanistic view, the new model advances the old one.

b) Analysis of a global SECIRS-type model using wastewater data

Given the complexity of our proposed model, it is important to show the advancements on the particular elements separately. Therefore, we consider the results of the immune-waning model separately from the impact of the mobility scheme and use a single (waning immunity) model for the whole of Germany over a two years time frame (2022-2024). The parameters used in this analysis differ slightly from those used in the spatially resolved model (Table 3 and Table 2) since we homogeneously aggregate heterogeneous immunity dynamics over the whole of Germany. We prevented from tweaking parameters too much to improve the global fit, as inferred parameters would necessarily be wrong on a local scale. We use wastewater data as an unbiased estimator to adequately capture disease dynamics in times of changing or absent testing strategies. From Fig. 2, we see that the new model qualitatively represents the three major surges and declines over the two year period indicated by the wastewater samples from the AMELAG [7]. On the other hand, the old model wrongly results in an eradiction of the virus in the population. We furthermore see that the development of reported cases diverges significantly from the wastewater measurements. We see that the global model does not fit well several local peaks induced by a heterogeneous spread of COVID-19 in Germany, see also the local wastewater measurements in the Appendix. Additional considerations to use and validate our novel model in different scenarios will be the focus of future work.

Table 2: **Model parameters for the global SECIRS-Type model without spatial resolution.** The table shows the basic transmission probability ($\rho_i^{(0)}$) and the increased protection ($p_{E_{PI}}$ and $p_{E_{II}}$) for individuals with higher immunity.

**Parameter**	**Age Group**					
	0-4	5-14	15-34	35-59	60-79	80+
$\rho_i^{(0)}$	$[0.06, 0.12]$	$[0.15, 0.21]$			$[0.24, 0.3]$	$[0.3, 0.4]$
$p_{E_{PI}}$	0.4					
$p_{E_{II}}$	0.4					

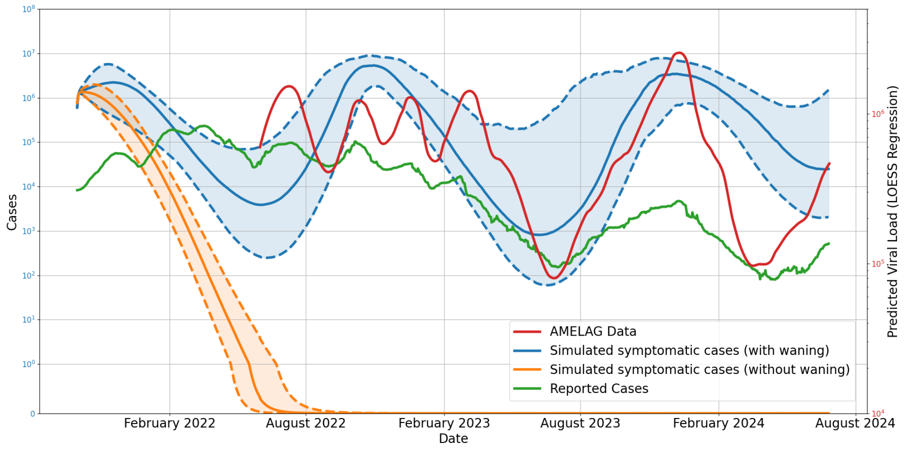

Figure 2: **Comparison of the simulated and reported cases with wastewater measurements over a two-year period (2022-2024).** New symptomatic infected individuals simulated with novel immune-waning model (blue) and existent no-waning model (orange). Obtained AMELAG wastewater data (red) and reported cases (green). The solid line represents the median of the simulation results, while the shaded area indicates the 25th to 75th percentile.

c) Furthermore, we validated our model on Post-Munich Oktoberfest 2022 (until the beginning of the winter season) and the propgation of mild and ICU cases over a period of several months.

Developments of Post-Oktoberfest until winter season 2022 in Bavaria

To further validate the effect of the novel mobility model, we simulate the Post-Munich Oktoberfest period until the beginning of the winter season 2022. The Oktoberfest, which took place from September 17 to October 3, 2022 and attracted around 5.7 million visitors [8]. Unfortunately, conducted tests have decreased significantly over 2022 and positive rate has been high, see [9] and Fig. 3, which makes reported case numbers quite unreliable and comparisons difficult. On an aggregated level we thus also include reported ICU numbers from [10]. We initialize the model based on the official reported data one week after the festival, at October 10 2022, and simulate until the end of the year using the parameters in Table 3. We adjusted the transmission probability ρ with a factor of 0.8. While the simulate mild cases largely exceed the reported cases (see Fig. 3 (left)), which is no surprise given the low number of tests and high positive rate, the simulated ICU admissions come close to the reported ICU admissions; see Fig. 3 (right).

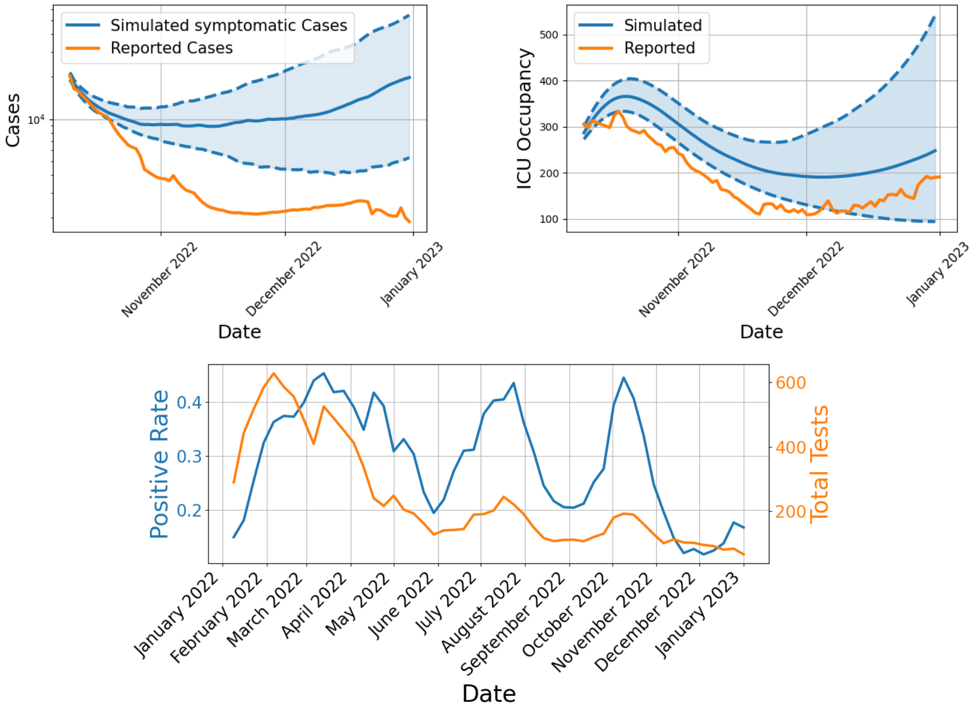

Figure 3: **Comparison of simulated and reported data for Bavaria and conducted tests.** The top left figure shows simulated daily new symptomatic against reported cases in Bavaria, with data smoothed using a 7-day moving average to reduce oscillations. The top right figure compares simulated ICU admissions to reported ICU data. The bottom figure presents the total number of tests conducted alongside with the positive test rate in Bavaria for the entire year 2022.

To provide spatial details of our simulations, we present the simulated number of symptomatic infections in a spatially resolved plot of Bavaria. In Fig. 4, we see the spread of infections across the federal state, with the numbers presented relative to the local population.

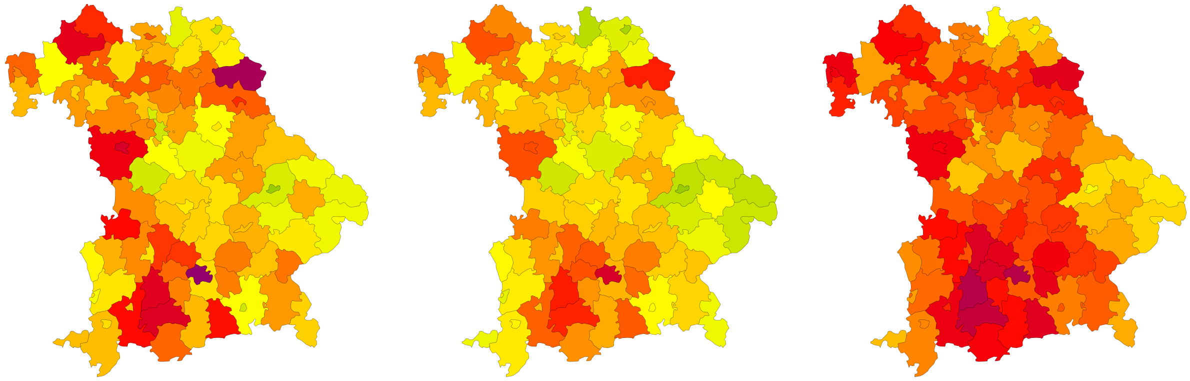

(a) October 31, 2022 (b) November 15, 2022 (c) December 1st, 2022

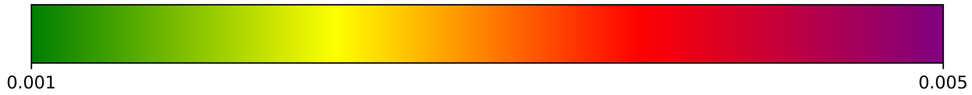

Figure 4: **Spatial distribution of symptomatic infections in Bavaria for selected days of the Post-Oktoberfest and beginning winter season 2022.** Presented values are provided relative to local population.

- *Next, I appreciate the combined setting of a late-phase epidemic, which is interesting. However, at this point, I find it hard to assess how this model is able to capture reality. I tried to make this assessment by interpreting Figure 11, which shows the green model curve (which should be close to the German reality during the time frame that was modelled, if I'm not mistaken) and 2 data curves (red and orange, where red corresponds to the DigiHero reports). Based on this Figure, I find it hard to see how well the red curve and the green curve match. Perhaps a rescaling of the DigiHero curve could help here, but perhaps it would be better to try to capture the DigiHero reporting dynamics in the model to allow for curves that are easier to compare? That being said, I understand that comparing symptomatic curves is not an easy thing to do (due to underreporting, as mentioned by the authors), in that regard, did the authors consider taking hospitalisations into account for evaluating the models (I would argue hospitalisation data has proven more stable during the course of the course of the pandemic)?*

  Answer: Regarding the DigiHero data, let us state that we have discussed with the study responsible the (potential) underdetection, as mentioned in the paper, although less prominent than in country-wide testing and case reporting. As for your suggestion to incorporate hospitalizations, we agree with the importance of using stable and reliable data for model evaluation. In Germany, hospitalization data has been subject to various reporting inconsis-

tencies. We think that ICU admission data (although not age-resolved as positive case data), as reported by the DIVI [10] quite reliably over the whole time of the pandemic, is the best metric available. We thus opted to validate the model further on ICU data.

For the symptomatic cases simulations as in the initial submission, we now also provide simulated ICU admissions and compare it to reported ICU data. In contrast to reported data, we see a substantial decrease over the first weeks of the simulation while the following dynamics are quite well fit.

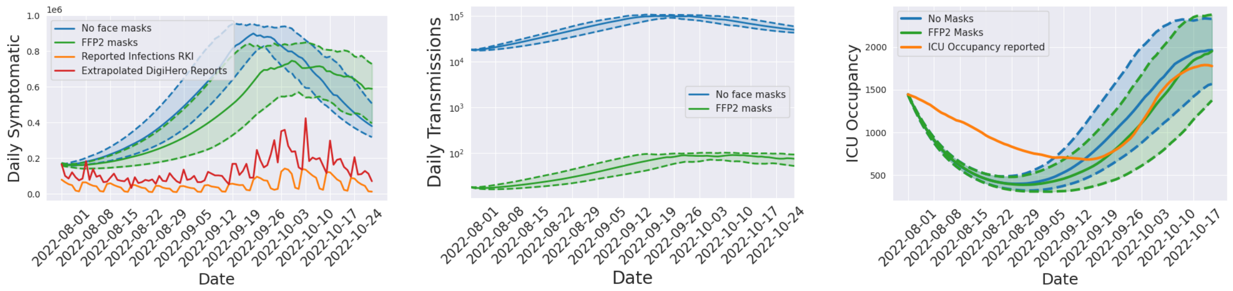

(a) Daily symptomatic infections. (Linear-scaled y-axis)

(b) Daily Transmissions in mobility. (Log-scaled y-axis)

(c) Simulated ICU occupancy compared with real data. (Linear-scaled y-axis)

Figure 5: **Daily number of symptomatic infections, transmissions and ICU occupancy.** The simulations provide results using correctly worn FFP2 masks (green) and no face masks (blue) in the mobility models. The number of daily symptomatic infections (left, blue and green) is compared to data provided by the DigiHero study (red), where the number of participants is extrapolated to the population in Germany and the official reported cases (yellow). Median values are indicated by the solid lines and shaded areas between the dotted lines represent the p25 and p75 percentiles of the particular ensemble runs. In the center plot, we visualize the daily transmission in the mobility models during the course of the simulation on a logarithmic scale. Furthermore, simulated ICU occupancy with different mask usage (right, blue and green) is compared to reported ICU occupancy (right, orange).

In addition to the figure, we also write: In order to validate the results obtained by our simulation, we compare to ICU occupancy in Germany from [10] which is a more stable and reliable indicator. In Fig. 5 (right), we see that our model is able to capture the reported ICU occupancy for the second part of the simulation very well while discharges on initially admitted are overestimated.

Furthermore, we provide a scenario for the aftermath of the Munich Oktoberfest 2022 until

the beginning of the winter seasons and where ICU developments are fit well over the whole time of simulation.

- *Regarding the presentation of the methods. It was not always easy to identify the authors' contributions given that the background and the novel methods are quite intertwined. I think it would be good to explicitly emphasise the contributions and add some more structure to the method section to help the reader. Perhaps it could help to split this section in a background section and a methods section, to make this more clear?*

  Answer: Thank you for your feedback. We understand the importance of clearly delineating the authors contributions. With the additionally introduced section on the theoretical properties of the novel model, we hope that new contributions are better recognized as generalizations of the previous model. We also think the the PLOS style, not allowing any section enumerations, complicates better references to particular subsections. However, to make the paper self-contained, we wanted to briefly repeat prior developments and directly contrast them to novel contributions. In order to separate background and novel contributions more clearly, we have, among other things, added the reference to the old paper to Fig. 3 and 4 and added at several occasions information on the novelty or the limitations of the old model.

- *Unless I missed it, the authors consider the type of travel contacts the same as contacts made at home, work, school. I would argue that contacts made during a travel (on the train/bus etc) are more distant, as individuals might not talk to each other. Earlier work in the context of influenza distinguished between conversational and physical contacts (https://journals.plos.org/ploscompbiol/ar but the types of contacts made at public transport might be considered to be of a different type? Can the authors comment on this, as assumptions in this regard could have an impact on some of the conclusions (e.g., Fig 11).*

  Answer: This is a very important point, we have been already considering for a long time. While agent-based models with local proximity definitions are promising additions to ODE-based models to correct for the fact of aerosol transmission, ODE-based models probably have to aggregate a multitude of effects in one or several variables. In our model, the contact rate parameter ϕ quantifies the number of contacts per person per day and does not rate the quality these contacts. However, more important for the quality of the contacts is the transmission probability from one contacted defined by ρ. Thus, ρ would be inherently suited to capture different types of contacts (i.e., conversational/physical), though it remains a simplified representation. However, classic contact surveys distinguishing between "conversational" (sometimes defined as "three or more words exchanged") and physical, do often not provide more information on the type of particular contact and a conversation lasting for some time could result in higher transmission risk than a limited physical contact. Within local contact settings such as "School", there is also an aggregation of diverse contact types like (close) interactions to seating neighbors and also more distant ones (such as those

across the classroom). We feel that a disaggregation of contact types would also involve (potentially bold) assumptions. However, the inclusion of (aggregated) aerosol transmission in (mean-field) models remains an open reserach question that also will drive our future research.

We have added the following summarizing remarks to the section *Epidemiological parameters for SARS-CoV-2 Omicron variant BA.1/2*: Theoretically, the transmission probability ρ could be chosen differently for the mobility model and the local contact model to account for varying interaction qualities. However, due to the aggregated view of ODE-based models and limited data availability, we decided to use the same value across both models, thereby assuming an identical transmission probability in both models.

- *Regarding reproducibility, the authors state "The total number of produced simulation data sums up to 200 GB and cannot be uploaded easily.". I would argue that the simulation results are not that important, but the code and data sources needed to reproduce these results are key. Can the authors explain whether this is possible with the material that is available on GitHub?*

  Answer: With the data sources provided by Robert Koch Institute, DIVI, destatis etc. (which can be downloaded and transformed in a simple way by the MEmilio epidata package to be used with our C++ models) and the open source code of MEmilio, all simulation results can be reproduced. While individual runs can differ due to different realizations of the random number generator (RNG), ensemble run results and percentiles (as presented in the paper) should be stable across different states of the RNG. As the novel models are not yet integrated in the main branch, development branches have to be pulled for production. We added the following to the Data availability section: The code and the different models are available on different branches. The introduced SECIRS-type model with waning immunity can be found in branch *323-waning-immunity-model.* The novel mobility model is available in branch *385-mobility-scheme-with-transmission-and-traveltimes. The combination of both branches and the simulations presented in this paper are available on branch paper-zunker-simulations-2024.*

Some minor remarks:

- although fighting infectious diseases → sounds strange, consider rephrase
  Answer: We thank the reviewer for attentive reading. We now write: Despite ongoing efforts to tackle infectious diseases,

- situations of high transmission (risk) → situations of high transmission
  Answer: We thank the reviewer for attentive reading. This has been corrected.

- Over the last years, to predict SARS-CoV-2 development in 19 Germany, contributions have been made by a variety of different approaches. -¿ Rephrase: Over the last years, contributions have been made by a variety of different approaches, to predict SARS-CoV-2 development in 19 Germany."

  Answer: We thank the reviewer for attentive reading. This has been changed.

- In the introduction, the authors provide a list of different types of models. I have two comments. First, I would split between agent-based and individual-based models. Second, the authors only seem to mention German models, but I think for the individual-based models, models like COVASIM and "STRIDE COVID-19" should also be mentioned? Same remark for the other model categories.

  Answer: Given the ample literature on infectious disease models and COVID-19 applications, we feel that we can only provide a non-exhaustive overview in one particular paper. To provide a, to some extent, complete list of different models and model groups, we had decided to lay a particular focus on Germany, where also our application is. However, we are happy to extend this section a bit more to also include the broader (yet non-exhaustive) perspective on important international models.

  We now write: From the international community, an even ampler set of models has been suggested to mitigate the spread of SARS-CoV-2. Some highly recognized international models can be found in [11, 12, 13, 14].

- Mathematical models based on systems of ordinary differential equations (ODE) → Mathematical models based on systems of ODEs (abbreviation was already introduced)

  Answer: We thank the reviewer for attentive reading. This has been corrected.

- When first reading through equation (1), I was confused that there was a patch notation (k), which it concerns a definition that does not consider a metapop approach yet. I think it would be more clear to first introduce a normal SIR model.

  Answer: We have also discussed this issue several times before finalizing and submitting the paper. We have decided to directly use the additional patch-superindex to not introduce the SIR model twice and to make the patch presentation perfectly consistent with the previous definitions. After introducing the model, we write: "For the sake of a consistent development of the new model, we have already introduced the index k that will later be used to refer to a particular region k but which is without meaning for the presentation in this subsection.". We agree with the reviewer that this might be confusing first, but we have intentionally decided to use a consistent notation throughout the paper and have therefore already used the k here.

- although the model is a pure infection dynamics model, just parameterized for mobility settings." → During the first read, this was hard to understand, consider a rephrase.

  Answer: We thank the reviewer for attentive reading. This has been corrected by writing: "In the following and for the sake of simplicity, we will refer to the mobility-based infection model simply as *mobility model* or *traffic model*. Note that this model basically is a pure infection dynamics model but is specifically parametrized to represent mobility settings"

- while only a portion $p_{tr}^{(k)} \in [0, 1]$ also has contacts in traffic locations (which is nontrivial as people might not" → don't see why this is nontrivial, I believe it is trivial that only a portion has contacts during travelling? Or do I misunderstand the point of the authors?

  Answer: Unfortunately, our wording might have been unclear. Our aim was to emphasize that the contact structure in transport is nontrivial, as some people may not commute at all or only use individual modes of transport (single persons cars, scooters, bicycles, ...) that do not involve contact with others. We have clarified the text accordingly: Without loss of generality, let us assume that all individuals have contacts in nontraffic locations (which is trivial, i.e., in home or work). However, only a subset $p_{tr}^{(k)} \in [0, 1]$ of individuals also have contacts in traffic locations, as some people may not commute at all or may use private transportation modes such as cars without additional passengers such that they do not come into contact with others (on commuting itself).

- we need to determine who is commuting at all. → not clear, rephrase
  Answer: We have rephrased the sentence to make this clearer: To accurately model mobility, it is crucial to first identify which individuals are participating in commuting activities.

- A important challenge
  Answer: We thank the reviewer for attentive reading. This has been corrected.

- where neither infection nor vaccination promises lasting protection → where neither infection nor vaccination *establishes* lasting protection
  Answer: We thank the reviewer for attentive reading. This has been corrected.

- "larger numbers of infected" → "larger numbers of infected individuals" (this way of writing is also used in the rest of the manuscript, so I would consider changing it there as well)
  Answer: We thank the reviewer for attentive reading. This has been corrected.

- The authors indicate that one variable was fitted (Table 3), but I did not find any additional explanation, unless I missed it.
  Answer: We agree that there is a lack of detailed explanation regarding the fitting process. The values used were initially derived from the reference provided in [1]. Subsequently, these values were manually fitted in a heuristic manner to achieve a closer match with the observed data. We now write in the caption of Table 3: The parameter for the baseline transmission probability was manually fitted with the observed data, starting from the findings in [1].

- number is ridiculously large, → rephrase
  Answer: We thank the reviewer for attentive reading. We now write "immense" instead of "ridiculously large".

- (here denoted Existing method, → missing a closing)
  Answer: We thank the reviewer for attentive reading. This has been corrected.

- "Existing method" → perhaps a more descriptive name of the original model would be more clear
  Answer: We agree with the reviewer, that the naming is not optimal. To address this, we have changed to more descriptive terms. The term "existing model" has been replaced with "Instantaneous Travel Method," and the novel scheme is now referred to as the "Travel Time Aware Method".

  As an example, we now write:

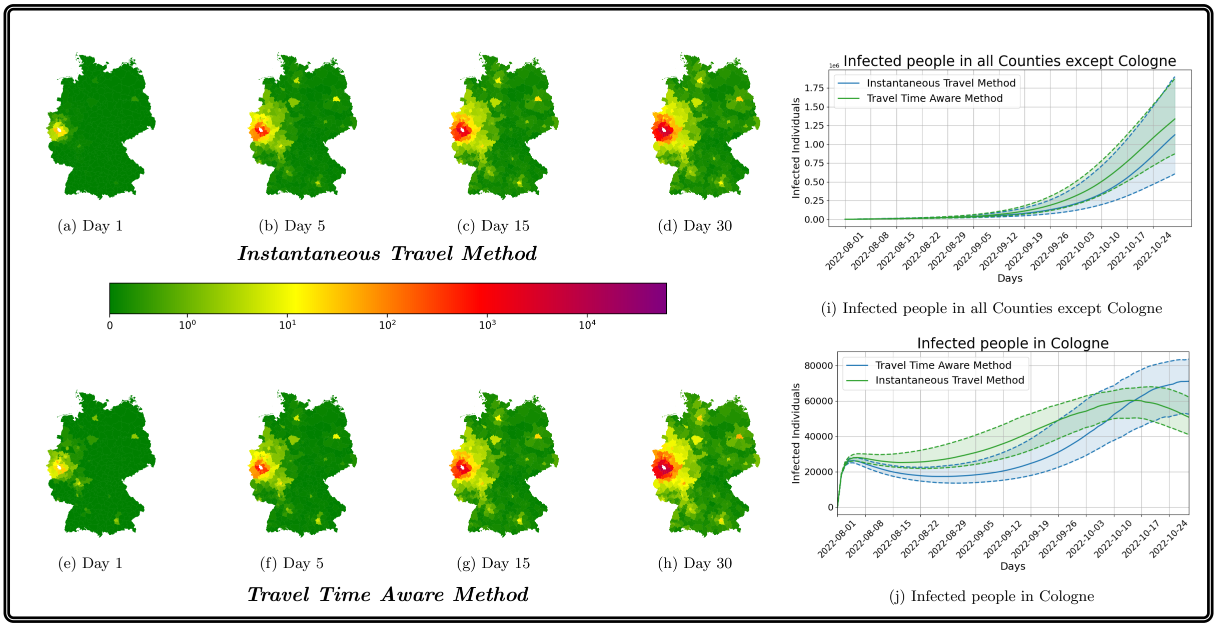

References

[1] Kühn MJ, Abele D, Mitra T, Koslow W, Abedi M, Rack K, et al. Assessment of effective mitigation and prediction of the spread of SARS-CoV-2 in Germany using demographic information and spatial resolution. Mathematical Biosciences. 2021; p. 108648. doi:https://doi.org/10.1016/j.mbs.2021.108648.

[2] Kühn MJ, Abele D, Binder S, Rack K, Klitz M, Kleinert J, et al. Regional opening strategies with commuter testing and containment of new SARS-CoV-2 variants in Germany. BMC Infectious Diseases. 2022;22(1):333. doi:10.1186/s12879-022-07302-9.

[3] Koslow W, Kühn MJ, Binder S, Klitz M, Abele D, Basermann A, et al. Appropriate relaxation of non-pharmaceutical interventions minimizes the risk of a resurgence in SARS-CoV-2 infections in spite of the Delta variant. PLOS Computational Biology. 2022;18(5):e1010054. doi:10.1371/journal.pcbi.1010054.

[4] Lange B, Jäger V, Rücker V, Hassenstein M, Harries M, Berner R, et al.. Interimsanalyse des IMMUNEBRIDGE-Projektes zur Kommunikation von vorläufigen Ergebnissen an die Modellierungskonsortien der BMBF-geförderten Modellierungsplattform; 2022. Available from: `https://doi.org/10.5281/zenodo.6968574`.

[5] Lange B, Jaeger VK, Harries M, Rücker V, Streeck H, Blaschke S, et al. Estimates of protection levels against SARS-CoV-2 infection and severe COVID-19 in Germany before the

2022/2023 winter season: the IMMUNEBRIDGE project. Infection. 2024;52(1):139–153. doi:10.1007/s15010-023-02071-2.

[6] Morris MD. Factorial Sampling Plans for Preliminary Computational Experiments. Technometrics. 1991;33(2):161–174. doi:10.1080/00401706.1991.10484804.

[7] Robert Koch-Institut F. Abwassersurveillance AMELAG; 2024. Available from: https://doi.org/10.5281/zenodo.13683260.

[8] Statista. Anzahl der Besucher auf dem Oktoberfest von 1980 bis 2023; 2024. Available from: https://de.statista.com/statistik/daten/studie/165511/umfrage/anzahl-der-besucher-auf-dem-oktoberfest-seit-1980/.

[9] Bayerisches Landesamt für Gesundheit und Lebensmittelsicherheit. Anzahl der Laboruntersuchungen auf SARS-CoV-2 in Bayern (Tabelle 11); 2023. Available from: https://www.lgl.bayern.de/gesundheit/infektionsschutz/infektionskrankheiten_a_z/coronavirus/karte_coronavirus/archiv2.htm.

[10] Deutsche Interdisziplinäre Vereinigung für Intensiv- und Notfallmedizin (DIVI). DIVI Intensivregister Tagesreport; 2022. Available from: https://www.divi.de/divi-intensivregister-tagesreport-archiv.

[11] Bershteyn A, Gerardin J, Bridenbecker D, Lorton CW, Bloedow J, Baker RS, et al. Implementation and applications of EMOD, an individual-based multi-disease modeling platform. Pathogens and Disease. 2018;76(5). doi:10.1093/femspd/fty059.

[12] Ferguson NM, Laydon D, Nedjati-Gilani G, Imai N, Ainslie K, Baguelin M, et al. Impact of non-pharmaceutical interventions (NPIs) to reduce COVID-19 mortality and healthcare demand. Imperial College; 2020. Available from: https://www.gov.uk/government/publications/impact-of-non-pharmaceutical-interventions-npis-to-reduce-covid-19-\mortality-and-healthca

[13] Willem L, Abrams S, Libin PJK, Coletti P, Kuylen E, Petrof O, et al. The impact of contact tracing and household bubbles on deconfinement strategies for COVID-19. Nature Communications. 2021;12(1). doi:10.1038/s41467-021-21747-7.

[14] Kerr CC, Stuart RM, Mistry D, Abeysuriya RG, Rosenfeld K, Hart GR, et al. Covasim: An agent-based model of COVID-19 dynamics and interventions. PLOS Computational Biology. 2021;17(7):1–32. doi:10.1371/journal.pcbi.1009149.
