# Supplementary material for: Novel travel time aware metapopulation models and multi-layer waning immunity for late-phase epidemic and endemic scenarios
Source: PLoS Comput Biol. 2024 Dec 16;20(12):e1012630. doi: 10.1371/journal.pcbi.1012630 (PMC11684649; doi:10.1371/journal.pcbi.1012630)
Supplement: Attachment — Submitted filename: answer_to_reviewer_2.pdf [file pcbi.1012630.s006.pdf]

Reviewer #2

I thank the authors for a very thorough and detailed revision, and congratulate them on this nice work. This revision addresses my comments and I only have a couple of minor remarks.

Answer: We highly appreciate the comments and proposed improvements the reviewers made to the original document. We are glad about the we were able to address your comments. In the following, we have addressed all the minor issues mentioned by the reviewer while highlighting changes in the manuscript accordingly.

1. "We use wastewater data as an unbiased estimator" → A bit strange to refer to data as an estimator? Moreover, I would not state that it is unbiased, cause that would be hard to show (also not convinced that it is). Perhaps rephrase as "We use wastewater as a proxy"?

   Answer: We admit that our previous sentence might have been unclear. Here, unbiased was meant as "without bias through potentially (changing) test strategies". We have rephrased this sentence and now write:

   "As changing or temporarily absent testing strategies lead to highly biased numbers of confirmed cases, we use wastewater data as a less biased proxy to adequately capture disease dynamics."

2. In Figure 4, the authors state "Spatial distribution of symptomatic infections in Bavaria". Given the revision that was done, perhaps it is better to show ICU cases her as well? And if this adjustment is made, perhaps give some information on how well these predictions follow the reported ICU data from a spatially perspective?

   Answer: That was also our original intention. However, we are faced with the problem that ICU patients are relocated across the counties as local capacity bottlenecks appear or in order to proactively avoid local capacity bottlenecks [1, 2]. On the other hand, even without transfers, hospitals often treat ICU patients from neighboring counties, e.g., in more urban areas where the capacities are low [3]. Additionally, in some cases specialized treatment (like in large university hospitals) was necessary, leading to patients to be admitted to surrounding counties. As of today, Oct 23, 2024, we see in the intensive care register[1] that from 893 clinics, roughly 50 % either do not provide information on ICU "high-care" (101/893; and potentially do not have), are "used to capacity" (210/893) or "have limited capacity" (215/893). Therefore, also DIVI-own visualization is on federal state only[2]. This means, while (ICU) predictions should be made based on local developments infection developments seen or validated in the wastewater, fitting of ICU should be done on federal state level.

[1] https://www.intensivregister.de/#/intensivregister

[2] https://www.intensivregister.de/#/aktuelle-lage/kartenansichten

3. The authors state ”In Fig. 5 (right), we see that our model is able to capture the reported ICU occupancy for the second part of the simulation very well while discharges on initially admitted are overestimated.” Can the authors add some discussion on the mismatch (reported vs model) between initially admitted ICU cases.

   Answer: That is a good point that we had missed to elaborate. As the average time spent on ICU varies with age [4, 5], age-resolved data would be best suited to adequately fit the models. However, provided ICU data is not age-resolved. This means that in different phases of the pandemic (or the endemic state), different ICU occupancy distributions are reported with the same number. While we try to extrapolate an age-resolved distribution from reported cases, there is certainly a different bias for different distributions, in particular, if the age distribution of reported cases develops differently than the (unknown) age distribution of ICU admissions. In future works, we will try to improve the initial state estimation, manual adaptation and fitting procedure to counter the different issues. We have added the following statement to the paper:

   ”Unfortunately, we could not resolve the mismatch in the beginning of the simulation period as ICU data is reported without age distributions, while average time spent on ICU varies with age [4, 5]. We here tried to extrapolate a demographic distribution of initial ICU occupancy using demographic distributions of reported cases. In future works, more advanced methods for initial state estimation will be developed.”

References

[1] Hentschker C, Mostert C, Klauber J, Malzahn J, Scheller-Kreinsen D, Schillinger G, et al. Stationäre und intensivmedizinische Versorgungsstrukturen von COVID-19-Patienten bis Juli 2020. Med Klin Intensivmed Notfmed. 2021;116(5):431–439. doi:10.1007/s00063-021-00776-6.

[2] Bundesamt für Bevölkerungsschutz und Katastrophenhilfe. Kleeblatt-Mechanismus; 2024. Available from: `https://www.bbk.bund.de/DE/Themen/Internationale-Angelegenheiten/Kleeblatt-Mechanismus/kleeblatt-mechanismus_node.html`.

[3] Deutsche Interdisziplinäre Vereinigung für Intensiv- und Notfallmedizin (DIVI). DIVI Intensivregister Tagesreport; 2022. Available from: `https://www.divi.de/divi-intensivregister-tagesreport-archiv`.

[4] Tobin RJ, Wood JG, Jayasundara D, Sara G, Walker CR, Martin GE, et al. Real-time analysis of hospital length of stay in a mixed SARS-CoV-2 Omicron and Delta epidemic in New South Wales, Australia. BMC Infectious Diseases. 2023;23(1):28. doi:10.1186/s12879-022-07971-6.

[5] Kühn MJ, Abele D, Mitra T, Koslow W, Abedi M, Rack K, et al. Assessment of effective mitigation and prediction of the spread of SARS-CoV-2 in Germany using demographic information and spatial resolution. Mathematical Biosciences. 2021; p. 108648. doi:https://doi.org/10.1016/j.mbs.2021.108648.
